# Dissecting the expression landscape of RNA-binding proteins in human cancers

**DOI:** 10.1186/gb-2014-15-1-r14

**Published:** 2014-01-10

**Authors:** Bobak Kechavarzi, Sarath Chandra Janga

**Affiliations:** 1 Department of Biohealth Informatics, School of Informatics and Computing, Indiana University – Purdue University, 719 Indiana Ave Ste 319, Walker Plaza Building, Indianapolis, IN 46202, USA; 2 Center for Computational Biology and Bioinformatics, Indiana University School of Medicine, 5021 Health Information and Translational Sciences (HITS), 410 West 10th Street, Indianapolis, IN 46202, USA; 3 Department of Medical and Molecular Genetics, Indiana University School of Medicine, Medical Research and Library Building, 975 West Walnut Street, Indianapolis, IN 46202, USA

## Abstract

**Background:**

RNA-binding proteins (RBPs) play important roles in cellular homeostasis by controlling gene expression at the post-transcriptional level.

**Results:**

We explore the expression of more than 800 RBPs in sixteen healthy human tissues and their patterns of dysregulation in cancer genomes from The Cancer Genome Atlas project. We show that genes encoding RBPs are consistently and significantly highly expressed compared with other classes of genes, including those encoding regulatory components such as transcription factors, miRNAs and long non-coding RNAs. We also demonstrate that a set of RBPs, numbering approximately 30, are strongly upregulated (SUR) across at least two-thirds of the nine cancers profiled in this study. Analysis of the protein–protein interaction network properties for the SUR and non-SUR groups of RBPs suggests that path length distributions between SUR RBPs is significantly lower than those observed for non-SUR RBPs. We further find that the mean path lengths between SUR RBPs increases in proportion to their contribution to prognostic impact. We also note that RBPs exhibiting higher variability in the extent of dysregulation across breast cancer patients have a higher number of protein–protein interactions. We propose that fluctuating RBP levels might result in an increase in non-specific protein interactions, potentially leading to changes in the functional consequences of RBP binding. Finally, we show that the expression variation of a gene within a patient group is inversely correlated with prognostic impact.

**Conclusions:**

Overall, our results provide a roadmap for understanding the impact of RBPs on cancer pathogenesis.

## Background

RNA-binding proteins (RBPs) have been identified as key regulatory components interacting with the RNA within a cell. Their function is largely dependent on their expression and localization within a cell. They may be involved in processes ranging from alternative splicing to RNA degradation. Combining together, RBPs form dynamic ribonucleoprotein (RNP) complexes, often in a highly combinatorial fashion that can affect all aspects of the life of RNA [1-3]. Due to their central role in controlling gene expression at the post-transcriptional level, alterations in expression or mutations in either RBPs or their binding sites in target transcripts have been reported to be the cause of several human diseases such as muscular atrophies, neurological disorders and cancer (reviewed in [4-7]). These studies suggest there is precise regulation of expression levels of RBPs in a cell. In fact, a recent system-wide study of the dynamic expression properties of yeast RBPs showed that RBPs with a high number of RNA targets are likely to be tightly regulated, since significant changes in their expression levels can bring about large-scale changes in the post-transcriptional regulatory networks controlled by them [8]. RBPs have also been shown to autoregulate their expression levels. Fluctuations in the expression of autoregulatory RBPs are significantly decreased [9]. These results show that a low degree of expression noise for RBPs is a characteristic feature of their normal state.

Cancer is a complex genetic disease and many of its regulatory factors have been identified as being irregularly expressed. In particular, changes in the normal expression of RBPs have been shown to alter their function leading to a cancer phenotype [10]. Enhanced eIF4E and HuR expression levels have been implicated in initiating translation of mRNAs encoding mostly for pro-oncogenic proteins and other cancer-promoting processes. For instance, Sam68 regulates the alternative splicing of cancer-related mRNAs [10]. Yet another example is the cell-specific alternative splicing of FAS (Fas cell surface death receptor, a member of the TNF receptor superfamily) mRNA. This has been linked to cancer predisposition depending on whether the pro- or anti-apoptotic protein form is produced as a result of the interplay between various RBPs on the FAS transcript [11-14]. In some cases, disruption of the functionality of RBPs, although without directly acting on oncogenic genes, has been shown to affect alternative splicing regulation or the regulation of alternative cleavage mechanisms on transcripts, which can lead to the development of cancer [15,16].

In a recent study, Castello and co-workers [17] utilized cross-linking and immunoprecipitation (CLIP) and photoactivatable-ribonucleoside-enhanced CLIP (PAR-CLIP) to isolate and validate, via proteomics, a set of approximately 850 high-confidence RBPs in humans. These approaches can be used to catalogue and study RBPs and their post-transcriptional networks in healthy and diseased states. By knowing the low degree of expression variation that is tolerated by RBPs in a healthy state and identifying them in mammalian systems, we can begin to investigate their dysregulation profiles in various disease conditions.

**Figure 1:**
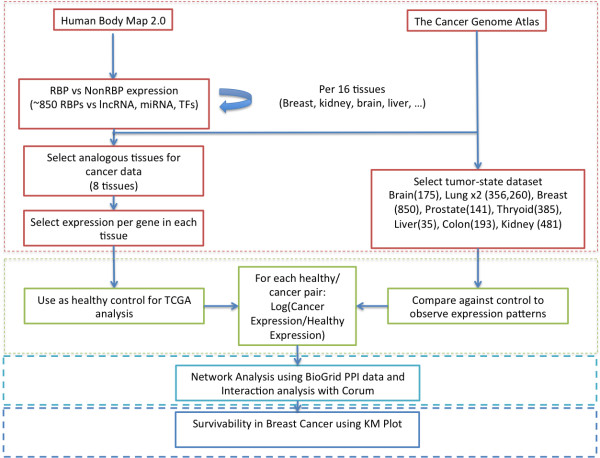
**Flow chart showing the different steps in the analysis of expression levels of RNA-binding proteins for human cancers.** The flow chart shows the acquisition and preparation of data (red), determination of patterns of dysregulation (green), network and interaction analysis (light blue), and survival analysis (dark blue). KM, Kaplan–Meier; lncRNA, long non-coding RNA; PPI, protein–protein interaction; RBP, RNA-binding protein; TCGA, the Cancer Genome Atlas; TF, transcription factor.

In this study, we analyzed the expression patterns of RBPs in a set of 16 healthy human tissues and compared their fold change in expression levels in nine human cancers using the high-resolution expression profiles based on RNA sequencing (RNA-seq) available from the Human BodyMap (HBM) [18] and the Cancer Genome Atlas (TCGA) [19] (see Figure 1, which outlines the different steps, and Materials and methods). We also compared the network properties of a set of 31 RBPs, which were found to be strongly upregulated (SUR) for most of the cancers studied. The network properties may help to determine the cause of the altered expression for the RBPs. Finally, a subset of RBPs was identified based on their expression profiles and network metrics and their contribution to the survival of patients with breast cancer was investigated.

## Results and discussion

### RNA-binding proteins show significantly higher expression than non-RNA-binding proteins and other regulatory factors for 16 human tissues

In eukaryotes, transcription and translation occur in different compartments. This gives a plethora of options for controlling RNA at the post-transcriptional level, including splicing, polyadenylation, transport, mRNA stability, localization and translational control [1,2]. Although some early studies revealed the involvement of RBPs in the transport of mRNA from the nucleus to the translation site, increasing evidence now suggests that RBPs regulate almost all of these post-transcriptional steps [1-3,20]. RBPs have a central role in controlling gene expression at the post-transcriptional level. Alterations in expression and mutations in either RBPs or their RNA targets (the transcripts that physically associate with the RBP) have been reported to be the cause of several human diseases, such as muscular atrophies, neurological disorders and cancer [4-6,21].

**Figure 2:**
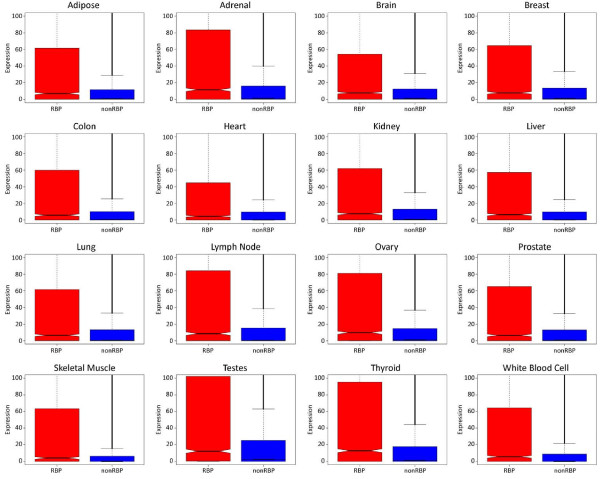
**Comparison of expression levels of RNA-binding proteins and non-RNA-binding proteins for 16 tissues from 80 healthy individuals studied in the Human BodyMap project.** Each of the 16 plots illustrates the significant differences in expression levels in RBPs (*P* < 2 × 10^-16^, Wilcoxon test) across adipose, adrenal, brain, breast, colon, heart, kidney, liver, lung, lymph node, ovary, prostate, skeletal muscle, testes, thyroid, and white blood cell tissues. The *x*-axis is the category of the observed factor and the *y*-axis is the expression level. RBP, RNA-binding protein.

Therefore, we first chose to study the mRNA expression levels of a repertoire of approximately 850 experimentally determined RBPs for all 16 human tissues for which expression data are available from the Human BodyMap 2.0 Project [18,22] (see Materials and methods). This analysis clearly showed that RBPs are significantly more highly expressed (*P* < 2 × 10^-16^, Wilcoxon test) than non-RBPs in all of the tissues (Figure 2). Closer inspection of the trends also revealed that some tissues, such as those from the testes, lymph and ovary, had particularly high RBP expression compared to non-RBPs. To determine the regulatory effect of RBPs at the post-transcriptional level compared to other regulatory factors, such as transcription factors (TFs), microRNAs (miRNAs) and long non-coding RNAs (lncRNAs), their expression levels were compared for different human tissues (see Additional file 1: Figure S1, Additional file 2: Table S1 and Materials and methods). This analysis further revealed that the expression levels of RBPs are significantly different for these 16 tissues compared to these families of regulatory factors (*P* < 2 × 10^-16^, Kruskal–Wallis test). Further analysis to compare the expression levels of RBPs and TFs across tissues revealed that except for the heart, kidney, ovary and testis, RBPs are significantly more highly expressed than TFs (*P* < 0.05, Wilcoxon test) (Additional file 2: Table S1). These observations suggest that in most tissues, the magnitude of expression of RBPs is more prominent than even TFs, possibly indicating their central role in controlling gene expression than previously anticipated. Our observation that RBPs are not significantly more highly expressed than TFs in heart, kidney and gonadal tissues like the testis and ovary suggests that both transcriptional and post-transcriptional regulators are equally important in terms of their expression levels in these tissues. In contrast, tissues like the liver (*P* < 3.57 × 10^-11^, Wilcoxon test) and white blood cells (*P* < 3.85 × 10^-5^, Wilcoxon test) were found to have significantly higher expression for RBPs compared to TFs, possibly indicating the importance of post-transcriptional regulation in the regenerative capabilities of a tissue or in monitoring inflammation and immune response.

The fact that RBPs exhibit a particularly high level of expression in some tissues suggests a need for extensive post-transcriptional control of gene expression in them. For example, the coordinated and cyclic processes of spermatogenesis in testes necessitate the essential temporal and spatial expression of pertinent genes [23]. In the human prostate, slight alterations to the androgen receptor functionality [24] or transcription factors [25] have been shown to lead to a cancerous state. These trends suggest that a significant fraction of the RBPome might play an important regulatory role in diverse human tissues, although in some gonadal and developed tissues, RBPs and TFs had similar levels of expression. Our results show that the high expression of RBPs is especially important in developmentally important tissues suggesting that any patterns of dysregulation could strongly effect these tissues [8].

### RNA-binding proteins are dysregulated across cancers and a subset are strongly upregulated across a majority of cancers

Based on our understanding of the expression landscape of RBPs in healthy human tissues, we next asked whether RBPs are dysregulated across cancers (see Materials and methods). Since expression data for healthy tissue was available for eight tissues from the Human BodyMap project corresponding to a set of nine different cancers profiled in the Cancer Genome Atlas (TCGA), we calculated the log-ratio of expression levels of RBPs in the healthy to cancerous states in each of the nine cancers (Materials and methods). Positive values represent a shift towards upregulation, or, more generally, increased transcript abundance. Negative log-ratios represent a trend of downregulation or decreased abundance. The log-ratio expression profile matrix for the nine cancers was hierarchically clustered to show patterns of similar dysregulation (Additional file 3: Figure S2 and Additional file 2: Table S1 includes log-ratio expression of RBPs). We observed that cancers in similar tissues (lung adenocarcinoma and lung squamous carcinoma) are clustered together suggesting a similar degree of dysregulation of the RBP repertoire. Our analysis also revealed that similar cancers, such as adenocarcinomas were clustered together. These trends indicate that expression ratios are reliable for profiling cancers with unique morphologies in various body locations.

**Figure 3:**
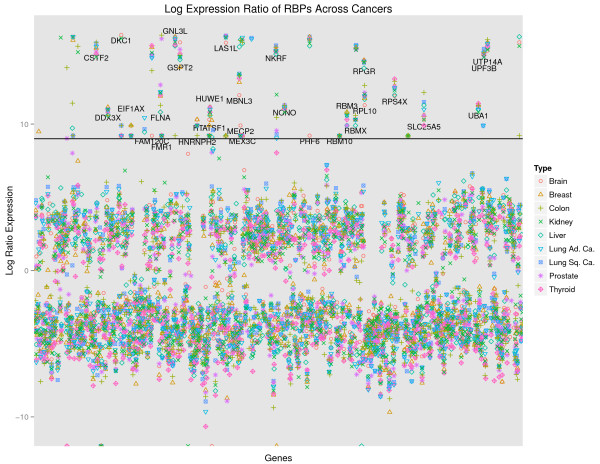
**Log-ratio of expression for cancer to healthy expression for RNA-binding proteins in nine human cancers.** The *x*-axis is an index of all the RNA-binding proteins that could be extracted from the expression data in the Cancer Genome Atlas. The *y*-axis is the ratio of the median expression level for each gene across patients versus the observed expression in the Human BodyMap data. Marked are the 31 strongly upregulated RBPs that have an expression ratio over nine across more than half of the studied cancers. Lung Ad. Ca., lung adenocarcinoma; Lung Sq. Ca., lung squamous carcinoma; RBP, RNA-binding protein.

An analysis of the log-ratios representing the fold changes in expression of RBPs between healthy and cancerous states for nine different cancers allowed us to define a criterion for classifying RBPs as strongly upregulated (SUR) or not (non-SUR) (Figure 3, Materials and methods). If an RBP, across six of the nine cancers, was found to have a log-ratio for expression level change of at least nine, it was classified as highly dysregulated, otherwise it was not considered to be a significantly dysregulated RBP. This also corresponded to the RBPs that belonged to the upper quartile of the fold changes in expression across cancers. According to this criterion, all the RBPs that had at least a ninefold change in expression were found to be only upregulated and hence this group was termed SUR RBPs (Figure 3). Table 1 lists these 31 SUR RBPs (Additional file 4: Table S2 provides detailed information).

**Table 1:** Strongly upregulated RNA-binding proteins identified from nine cancers in humans and their cancer relevant references

**Associated gene name**	**Description**	**References**
CCDC124	Coiled-coil domain containing 124	
CSTF2	Cleavage stimulation factor, 3′ pre-RNA, subunit 2, 64 kDa	[26]
DDX3X	DEAD (Asp-Glu-Ala-Asp) box polypeptide 3, X-linked	[27-29]
DKC1	Dyskeratosis congenita 1, dyskerin	[30-32]
EIF1AX	Eukaryotic translation initiation factor 1A, X-linked	
FAM120C	Family with sequence similarity 120C	
FLNA	Filamin A, alpha	[33-36]
FMR1	Fragile X mental retardation 1	
GNL3L	Guanine nucleotide binding protein-like 3 (nucleolar)-like	[37,38]
GSPT2	G1 to S phase transition 2	
HNRNPH2	Heterogeneous nuclear ribonucleoprotein H2 (H')	
HTATSF1	HIV-1 Tat specific factor 1	
HUWE1	HECT, UBA and WWE domain containing 1, E3 ubiquitin protein ligase	[39]
LAS1L	LAS1-like (*Saccharomyces cerevisiae*)	
MBNL3	Muscleblind-like splicing regulator 3	
MECP2	Methyl CpG binding protein 2 (Rett syndrome)	
MEX3C	Mex-3 homolog C (*Caenorhabditis elegans*)	
NKRF	NFKB repressing factor	[40]
NONO	Non-POU domain containing, octamer-binding	[41,42]
PHF6	PHD finger protein 6	[43-45]
RBM10	RNA-binding motif protein 10	
RBM3	RNA-binding motif (RNP1, RRM) protein 3	[46-49]
RBMX	RNA-binding motif protein, X-linked	[50]
RBMX2	RNA-binding motif protein, X-linked 2	
RPGR	Retinitis pigmentosa GTPase regulator	
RPL10	Ribosomal protein L10	
RPS4X	Ribosomal protein S4, X-linked	
SLC25A5	Solute carrier family 25 (mitochondrial carrier; adenine nucleotide translocator), member 5	
UBA1	Ubiquitin-like modifier activating enzyme 1	[51,52]
UPF3B	UPF3 regulator of nonsense transcripts homolog B (yeast)	
UTP14A	UTP14, U3 small nucleolar ribonucleoprotein, homolog A (yeast)	

We then asked whether tumor-matched normal expression data for TCGA samples can further support the set of SUR RBPs identified here. Although ‘normal’ site tissue samples from TCGA cannot provide an adequate control, since these samples are collected from a cancerous tissue and it is entirely feasible that the expression levels would still be in a state of dysregulation at the neighboring sites, this analysis can still provide an additional level of support for SUR RBPs. Additionally it is not possible to control for morphological types of tumors, which, depending on their type, can affect more than just the site of the tumor growth. Nevertheless, we profiled the tumor-matched normal expression levels that are available for eight of the nine cancer types with varying number of samples for breast (106 patients), colon (20 patients), kidney (69 patients), liver (49 patients), two types of lung cancers (57 and 50 patients), prostate (45 patients) and thyroid (58 patients). As suspected, we found the fold changes in expression for all the genes across eight cancers to be minimal (median [IQR] 0.055 [-0.28-0.39]), suggesting that tumor-matched normal expression data may not reflect a true healthy control. However, when we compared the fold changes in expression levels for RBPs and non-RBPs in the tumor-matched samples across cancers, we found that RBPs exhibited significantly higher fold changes compared to non-RBPs (median [IQR] 0.104 [-0.07:0.29] for RBPs versus median [IQR] -0.034 [-0.39:0.25] for non-RBPs, *P* < 2.2 × 10^-16^, Wilcoxon test) clearly indicating that RBPs are still significantly upregulated in tumors.

Further analysis to test for the enrichment of RBPs in the top quartile of upregulated genes across cancers revealed that RBPs are strongly over-represented in this list (*P* = 1.62 × 10^-93^, hypergeometric test). We also found that all the SUR RBPs are significantly dysregulated (*P* < 0.001, *t*-test comparing tumor and matched normal samples) in at least four of the eight cancers profiled (Additional file 2: Table S1). When we raised the stringency to identify an RBP to be dysregulated in at least six or more cancer types, we still found 24 of the original 31 SUR RBPs to be detected at *P* < 0.001. Very few SUR RBPs from the cancer types Kidney renal cell carcinoma (KIRC) and Liver Hepatocellular Carcinoma (LIHC) were found to be significantly altered in the tumor-matched analysis. While most of the SUR RBPs were found to be upregulated in the tumor-matched analysis, we also found cases of downregulation (Additional file 2: Table S1). Nevertheless, SUR RBPs as a group were also found to be strongly over-represented in the top quartile of the upregulated set in the tumor-matched analysis (*P* = 2.16 × 10^-8^, hypergeometric test), further supporting the notion that SUR RBPs identified using an external healthy control across a broad range of cancers are a confident set of dysregulated RBPs.

Non-RBP log-ratios showing the expression changes were also calculated using the external healthy data to determine if the proportion of strongly upregulated genes (SURs) in RBPs is significantly enriched. We found that the proportions were significantly different (*P* < 0.05, hypergeometric test) with RBPs having a higher proportion of SURs than non-RBPs. Several of these SUR RBPs were annotated to function in important biological processes, such as regulation of gene expression, transcriptional regulation and transport of biomolecules, although very few studies have explored their role in the context of post-transcriptional control, suggesting that their functional roles are far more diverse than previously understood and appreciated.

Of these RBPs classified as SUR RBPs, we note several that have already been implicated in complex genetic disorders and cancer or in cellular regulation and proliferation (Additional file 4: Table S2). Identified RBPs, such as NONO, are involved in RNA biogenesis and DNA double-strand break repair, and have been found to be regulated by other factors, when dysregulated potentially promote carcinogenesis [41]. DDX3X, a member of the DEAD box RNA helicase family, has been shown to affect Wnt pathways, which leads to the developments of cancers [27]. DDX3X has also been demonstrated to promote growth and neoplastic transformation of breast epithelial cells [28]. Another SUR RBP, LAS1L was identified to interact with PELP1, which is implicated in pancreatic cancers [53]. HUWE1 is a member of the HECT family of E3 ubiquitin ligases, which has been identified as being overexpressed in breast, lung and colorectal cancers [54]. Indeed, increasing evidence now points to the role of novel ubiquitin-protein ligases in binding to RNA [55,56]. For instance, ubiquitin-like fold has been recently shown to be independently enriched in novel unconventional RBPs identified in the yeast genome [57]. The RNA-binding protein RBM3 is associated with cisplatin sensitivity, the probability of a patient becoming resistant to cisplatin treatment and a positive prognosis in epithelial ovarian cancer [46]. RBM3 has seldom been found expressed in normal tissues, but it is more expressed in common cancers, particularly for the nuclear expression of Estrogen-Receptor (ER) positive tumors. These findings suggest the possible utility of the gene as a positive prognostic marker [47,48].

PHF6 encodes a plant homeodomain (PHD) factor containing four nuclear localization signals and two imperfect PHD zinc-finger domains and it has been proposed that it has a role in controlling gene expression [58]. Inactivating mutations in PHF6 cause Börjeson-Forssman-Lehmann syndrome, a relatively uncommon type of X-linked familial syndromic mental retardation [58-60]. Recent studies show that mutations of this gene are implicated in the development of T-cell acute lymphoblastic leukemia and mutations have been detected in other forms of leukemia as well, suggesting a strong role in tumorigenesis [43,61]. For other nucleolar proteins such as dyskerin (DKC1), which is responsible for the biogenesis of ribonucleoproteins and telomerase stability, the loss or gain of functions is associated with tumorigenesis [30-32]. Filamin A (FLNA) is an actin-binding protein, which interacts with a number of proteins including signaling molecules and membrane receptors, and its expression has been correlated with metastases in prostate and lung cancers [33,34]. A recent study demonstrated the role of FLNA as a nucleolar protein that associates with the RNA polymerase I (Pol I) transcription machinery to suppress rRNA gene transcription [62]. Although further confirmation of how the global RNA-binding role of unconventional RBPs, like the E3 ubiquitin ligase HUWE1, contribute to cancer is needed, increasing evidence suggests that several enzymes and kinases bind to RNAs to control numerous cellular processes [57,63]. Recent genome-wide screens for novel RBPs further support these observations, suggesting that unconventional RBPs are enriched for enzymatic functions [57,64]. Functional enrichment analysis of SUR RBPs using the DAVID functional annotation system [65] revealed that RNA splicing, nucleotide binding and ribosome biogenesis were the common biological processes associated with these proteins, with a significant fraction of them associated with nucleolus and nuclear lumen cellular components (Additional file 4: Table S2).

Our observations combined with the existing corpus of literature in support of the roles for several of these SUR RBPs in cancerous states, suggest that their dysregulation could be the cause or result of the cancer phenotypes, especially given that even slight alterations in the expression levels of RBPs can bring about large-scale changes in the RBP–RNA interaction networks that they control [8]. It is important to note that although some of these SUR genes shown in Table 1 have been described in relation to cancer, there is little evidence in support of their contribution to either being RBPs or their post-transcriptional network as a contributing factor for the cancer phenotype. Our results in this study implicate them as a strongly upregulated set of RBPs across multiple cancers. Our analysis also corroborates that these significantly dysregulated RBPs are not an artifact of aberrations in calculations, or due to variability in patient expression data mainly because: (1) most of our patient sample sets are at least of the order of 100 for the cancers studied and (2) fold changes in expression levels between healthy and cancerous states for each patient were used to calculate the median fold change in expression of an RBP to account for extreme outliers. Our results also emphasize that these high expression levels may be indicative of a major dysfunction of these RBPs in addition to dysregulation. For example, the mutated form of PHF6, which is implicated in various forms of leukemia, has higher expression. Alternatively, the change in expression may be a result of an upstream alteration in the regulatory mechanisms, for example NONO; another example is that NKRF expression is regulated by miR-301a [40]. The high expression of some of these RBPs may be the result of their normal physiological levels being too low compared to a cancer context, as is the case for the proposed positive prognostic marker, RBM3. So a natural question to ask is whether RBPs have some prognostic impact for cancer, starting from the trends that have been observed in this expression analysis.

### Strongly upregulated and non-strongly upregulated RNA-binding proteins exhibit significantly different within-group path lengths and variability in expression is related to the number of interactions

**Figure 4:**
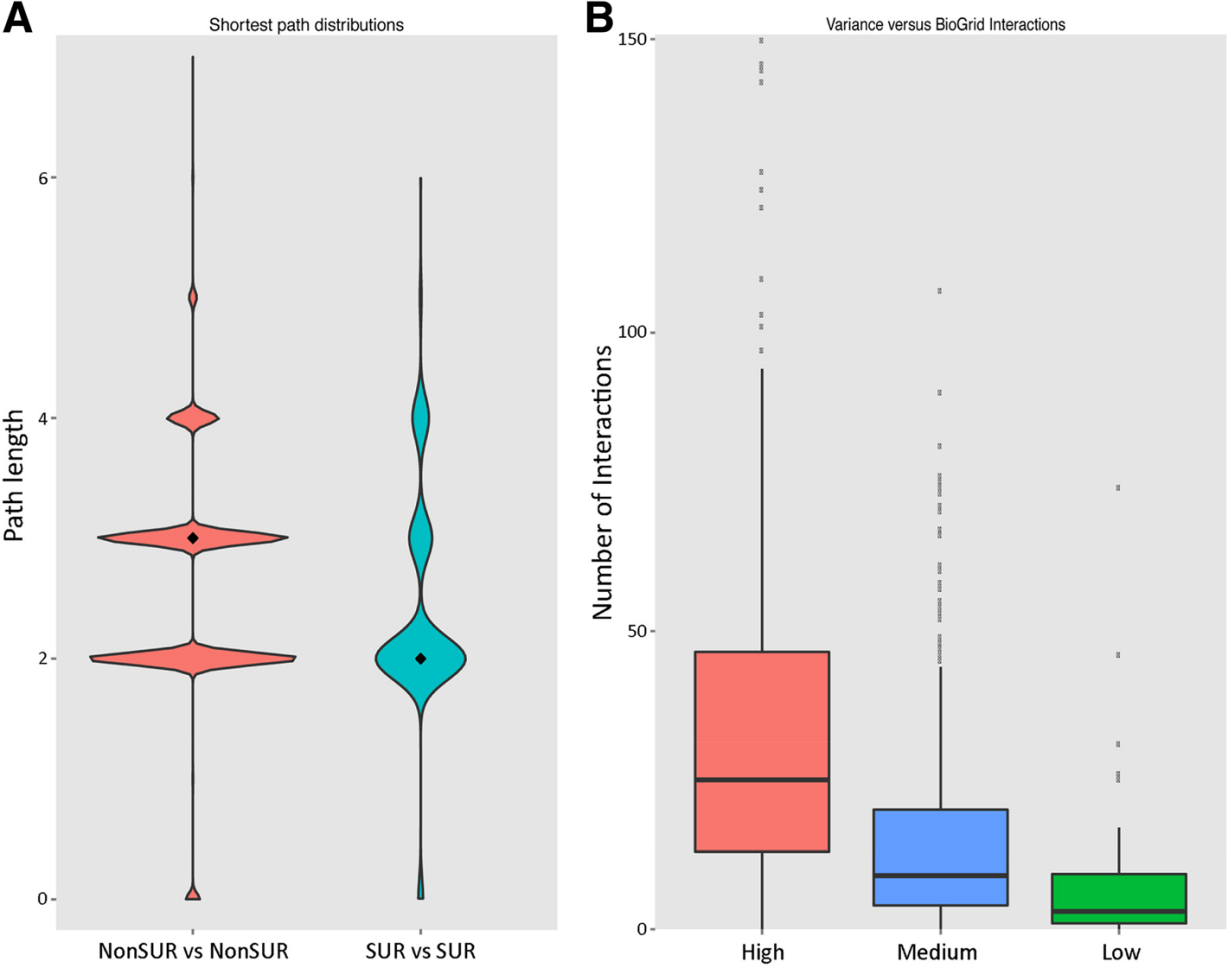
**Interaction profiles of RBPs. (A)** Distribution of shortest path lengths between every pair of RBPs belonging to SUR and non-SUR RBP groups using the protein–protein interactions documented in the BioGRID database [66], shown as violin plots. The width of each plot is the frequency distribution and the diamond is the median value for the category. SUR RBPs were found to have significantly shorter path lengths amongst themselves in comparison to non-SUR RBPs (*P* < 2 × 10^-16^, Wilcoxon test). **(B)** Box plot showing the number of interactions identified in BioGRID data for RBPs classified by variability levels defined by observed percentiles. The higher the variability for a RBP, the higher the observed number of protein interactions (*P* = 9.247 × 10^-16^, low vs medium; *P* < 2.226 × 10^-16^, low vs high; *P* = 6.6556 × 10^-16^, medium vs high, KS test). RBP, RNA-binding protein; SUR, strongly upregulated; Kolmogorov–Smirnov test (KS test).

To identify further characteristics that differentiate SUR RBPs in cancer, we calculated the network properties of all the RBPs using a network constructed from the experimentally reported set of protein–protein interactions in the human genome obtained from the BioGRID database [66] (see Materials and methods). In particular, we computed the shortest paths between pairs of proteins within SUR and non-SUR RBP groups (that is, distances from SUR RBPs to SUR RBPs and distances from non-SUR RBPs to non-SUR RBPs) (Figure 4A). SUR RBPs were found to have significantly shorter path lengths to each other when compared to non-SUR RBP path lengths (*P* < 2 × 10^-16^, Wilcoxon test). Other network metrics such as normalized degree distribution, normalized closeness, normalized betweenness and mean path lengths for RBPs in each group were also calculated (see Materials and methods). However, we found no significant difference between SUR and non-SUR RBPs for these properties (Additional file 5: Figure S3). This suggests that the interaction properties of an individual RBP (whether it is a hub and so on) do not relate to its dysregulation but rather the set of SUR RBPs are closely intertwined in the physical interaction network compared to the non-SUR RBPs. Although our observations on dysregulation are at the RNA level, it is possible to speculate, from the shorter path lengths observed, that the interaction network and crosstalk between SUR RBPs could also be perturbed in cancer genomes, with one or more of the SUR RBPs predominantly contributing to this perturbation.

Since our analysis of the shortest path lengths between RBPs from SUR and non-SUR groups suggested that the particular protein interaction partners of RBPs might play an important role in mediating or cascading the effect of dysregulation, we rationalized that the protein complex size and a RBP’s occurrence frequency in protein complexes would be related to their sensitivity to dysregulation. RBPs long have been known to form protein complexes, and if a key component within a complex is dysregulated or malformed, it would affect its overall functionality. If a SUR RBP was very prolific we would expect that many patterns of dysregulation would occur downstream as a result of the formation of a faulty complex. Furthermore, if these SUR RBPs participate in smaller complexes, it may be that their dysfunction will not be regulated or counteracted by other members within the complex. From the CORUM data [67] (see Materials and methods), five SUR RBPs were identified and 172 non-SUR RBPs were identified. We found that for the two classifications of RBPs (SUR vs non-SUR), there were no significant differences in distributions for either complex size or complex frequency nor was there any correlation with expression levels (Additional file 6: Figure S4 and Additional file 7: Figure S5). While the current coverage of the experimentally characterized human protein complexes is very limited, these results indicate that SUR and non-SUR RBPs do not have significant differences in terms of their protein complex membership.

We next asked whether the variability in expression levels of an RBP across cancer patients is different between SUR and non-SUR RBPs. To address this question, we choose breast cancer as our disease model due to the fact that it is the cancer with the most patient samples in TCGA and would naturally be the most robust dataset for identifying variation in the fold changes in expression levels of a RBP. We found that SUR and non-SUR RBPs did not exhibit significantly different expression variation (*P* = 0.1212, KS test), which was measured as the median absolute deviation (MAD) in the expression fold changes between healthy and cancerous tissue across all the patients (see Materials and methods). However, an analysis to test the relation between expression variation and the number of protein interactions of an RBP revealed that the higher the expression variation, the higher the number of protein interaction partners of the RBP (Figure 4B). Indeed, we noticed a significant difference in the number of interactions in the classified levels of variability for RBPs (*P* = 9.247 × 10^-16^, low vs medium; *P* < 2.226 × 10^-16^, low vs high; *P* = 6.6556 × 10^-16^, medium vs high, KS test). In contrast, TFs did not exhibit such significant differences in the number of interactions with the classified levels of variability (*P* = 0.8931, low vs medium; *P* = 0.0014, low vs high; *P* = 0.01, medium vs high, KS test). However, for non-RBPs a significant difference was found between medium and high as well as between high and low levels of variability (*P* = 0.7519, low vs medium; *P* < 2.2 × 10^-16^, low vs high; *P* < 2.2 × 10^-16^, medium vs high, KS test). The observation that the higher the variability in expression of a RBP the more interactions it has, suggests that fluctuating RBPs whose expression is not tightly controlled might have more promiscuous (non-specific) protein interactions (and protein complexes) thereby leading to RNA off-targets at post-transcriptional level. Our results also suggest that such dysregulation may be suppressed or is minimal due to the lower number of interactions for RBPs with less variability in expression. Our analysis here has focused on the RNA expression levels of RBPs though it is likely that there will be influences from diverse post-transcriptional regulatory phenomena like alternative splicing, translation control and post-translational modifications, which will affect the ultimate protein levels. Our observations do provide evidence that RBPs with high variability in expression have a higher number of protein interactions.

### Survival contributions of RNA-binding proteins in breast cancer is related to network proximity to strongly upregulated RBPs and variability in expression across patients

**Figure 5:**
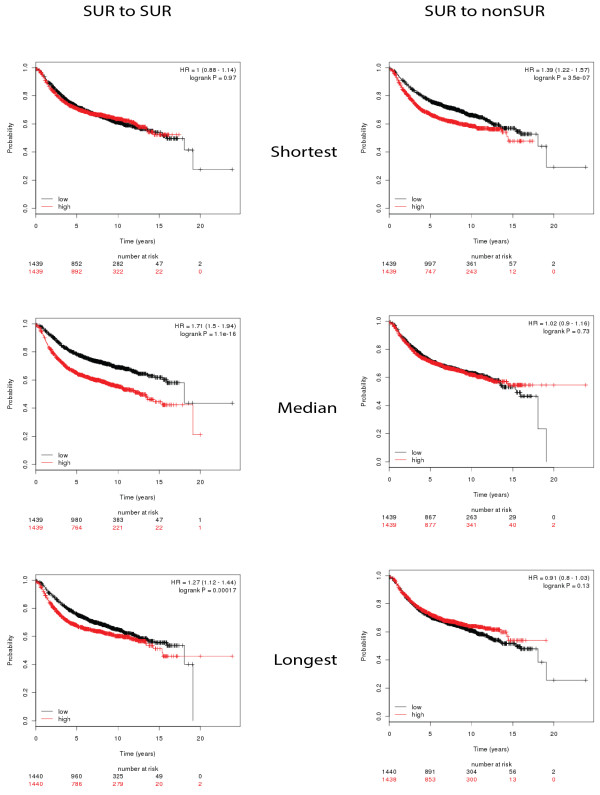
**Survival of patients with breast cancer for different expression levels and path lengths for within and between expression groups of RNA-binding proteins.** SUR (left) and non-SUR (right) survival for a sample of five RBPs classified by path length (shortest, median or longest). Curves in red are survival plots for patients with enhanced expression of the selected genes based on more than 1,800 patients’ expression profiles from the KM plot [68]. The within-group path ranking for SUR RBPs suggests that as the mean path lengths increase the contribution of the SUR RBPs in prognosis tends to increase. While between groups, RBPs having shorter path lengths to a SUR RPB contribute the most to prognosis. KM, Kaplan–Meier; RBP, RNA-binding protein; SUR, strongly upregulated; HR, Hazard Ratio.

Based on our observation that SUR and non-SUR RBPs significantly differ in their within-group shortest path lengths, we questioned whether the path length of an RBP within the protein–protein interaction network might contribute to its prognostic impact for a cancer. We ranked each RBP in each classification based on the mean path lengths to all connected nodes in the BioGRID protein interaction network and also computed the mean shortest paths to other nodes belonging to SUR RBPs and non-SUR RBPs. This allowed the construction of profiles for overall mean path lengths, lengths within-group for members of the SUR and non-SUR groups, and between the groups. The top five genes with the shortest and longest mean path lengths, and a randomly selected set of genes with intermediate mean path lengths, were selected for the survival analyses (Figure 5) (see Materials and methods). We found that as the mean path lengths between SUR RBPs increased, their contribution to prognostic impact increased. This suggests that SUR RBPs with longer path lengths, that is, those with higher network distances with respect to other SUR RBPs, are more likely to contribute independently to survival as they might influence a larger fraction of the dysregulated network of SUR RBPs. On the other hand, when non-SUR RBPs were sorted by rank based on their mean path lengths with respect to SUR RBPs, we found the opposite trend. This suggests that non-SUR RBPs with shorter distances to SUR RBPs contribute to the perturbation of an important section of the RBP protein interaction network. In particular, if a non-SUR RBP has a shorter path length, it has a good prognostic impact on survival for patients with breast cancer due to its lower expression. SUR RBPs are potentially in a malfunctioning state, and the closer a RBP is to them, the more the prognostic impact influenced by the SUR RBP interactions.

**Figure 6:**
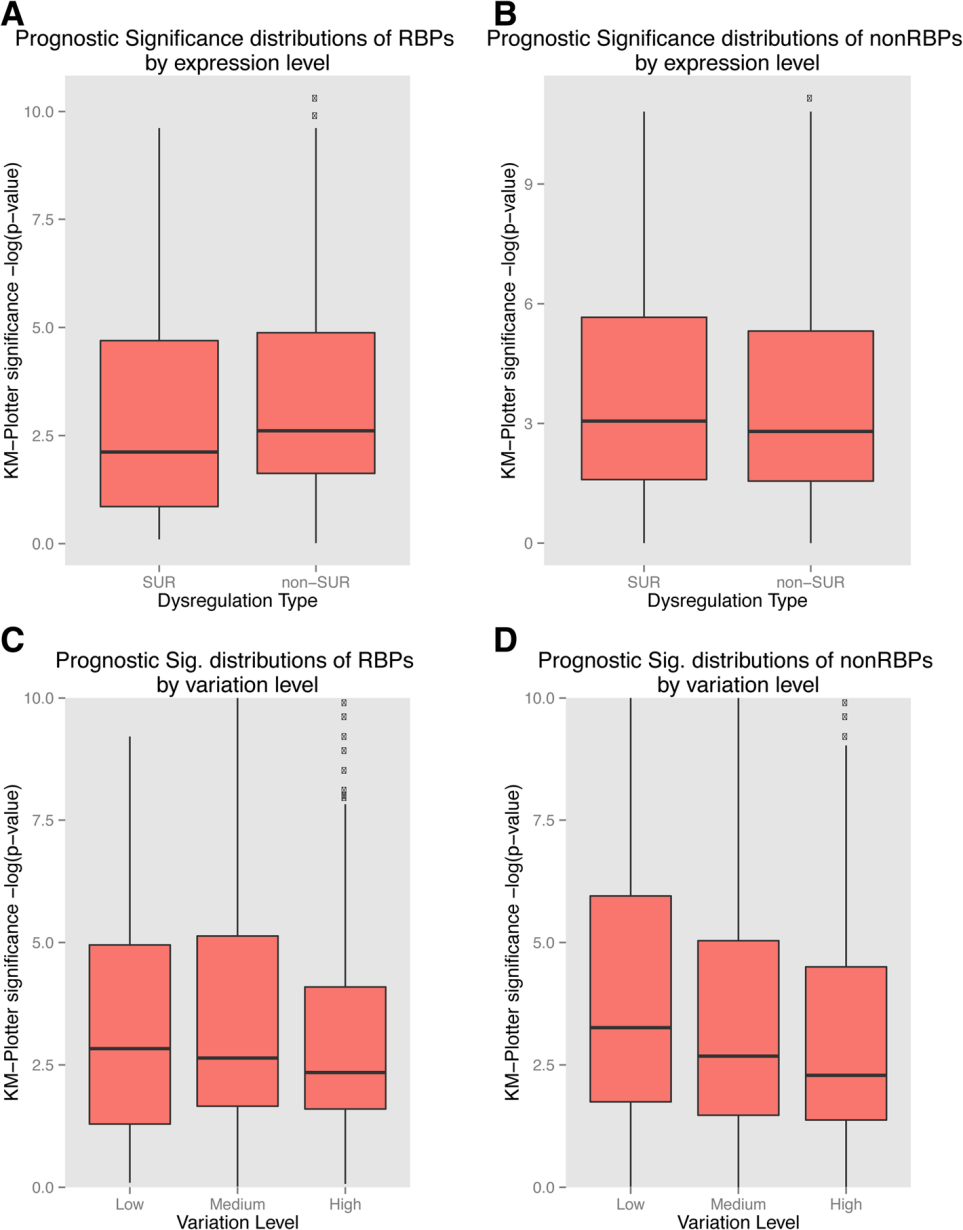
**Comparison and distribution of prognostic impact based on expression dysregulation and expression variability in breast tissue.** RNA-binding proteins **(A, C)** and non-RNA-binding proteins **(B, D)** were categorized based on their level of dysregulation as healthy or cancer expression (SUR or non-SUR) and the variability of expression levels (high, medium or low) in patients with breast cancer. The statistical significances for the differences in the distributions of prognostic impact are discussed in the main text. KM, Kaplan–Meier; RBP, RNA-binding protein; Sig., significance; SUR, strongly upregulated.

We then compared the overall significance of the Kaplan–Meier *P* values (-log[*P*]) for groups of RBPs classified by their level of dysregulation (SUR versus non-SUR) and their levels of variability in expression across patients (high, medium and low variability determined by quartiles, see Materials and methods) in breast cancer (Figure 6). We observed that for both RBPs and non-RBPs, there was no significant difference between SUR and non-SUR genes in terms of prognosis for survival (*P* = 0.12 and *P* = 0.06, KS test) (Figure 6A,B). However, when we compared the significance of the *P* values for survival between SURs from RBP and non-RBP groups we found them to be significantly different (*P* = 0.05, KS test). We noted that in the comparison between variability levels of genes in RBPs, there was no significant difference between the Kaplan–Meier (KM) analysis significance levels (*P* = 0.945, low vs medium; *P* = 0.3566, low vs high; *P* = 0.1478, medium vs high, KS test) (Figure 6C). For non-RBPs, we found that the levels of variability did have a very significant difference in the significance of KM-plotter survival *P* values (*P* < 2.226 × 10^-16^, low vs medium; *P* < 2.226 × 10^-16^, low vs high; *P* = 6.6556 × 10^-16^, medium vs high, KS test) suggesting that, in general, the higher the expression variation of a group of genes, the smaller is their contribution to prognosis for survival (Figure 6D). While there was no significant difference in RBPs we did observe a similar weak trend where the lower the variance in expression across patients, the greater the KM-plotter significance. A highly variable RBP has less effect on survival because it could potentially be regulated by a number of other factors and could be the result of an indirect effect, whereas low variability RBPs have a less but more direct effect on the prognosis for an individual and hence could be the actual drivers. This also corroborates our notion after observing variability versus the number of protein interactions (Figure 4B). More generally, our results suggest that while we observe a larger proportion of SUR RBPs, their elevated expression alone does not necessarily mean they have a direct effect on positive or negative prognoses.

## Conclusions

In this study, we investigated the gene expression profiles of RBPs in healthy humans for 16 tissues and found that RBPs are consistently and significantly highly expressed compared to other classes of genes (non-RBPs) as well as in comparison to well-documented groups of regulatory factors like transcription factors, miRNAs and lncRNAs. This, in concordance with previous research, emphasizes their importance in post-transcriptional regulatory control across all the tissues. To understand the expression profile changes in a disease state for hundreds of RBPs in the human genome, we obtained analogous RNA-sequencing-based expression data for a total of 2,876 patient samples spanning nine cancers from TCGA and calculated a log-ratio for expression between cancer and healthy states. We showed that there is a unique signature of approximately 30 RBPs that had significantly increased expression levels across six out of nine (two-thirds) cancers profiled. These could be clearly labeled as a set of SUR RBPs delineating them from the rest of the RBPs based on the change in expression levels. This proportion of SUR RBPs in the RBP population is greater than the proportion of SUR non-RBPs suggesting for the first time that the expression levels of a significant fraction of the RBPs are affected in cancerous states. Analysis of the protein–protein interaction network properties for SUR and non-SUR group of RBPs, suggested that the shortest path length distributions between SUR RBPs is significantly lower than that observed for non-SUR RBPs. This observation together with survival analysis based on path lengths suggests that not all the SUR RBPs might be directly implicated in cancer but rather that a cause-and-effect relation might hold between some of the SUR RBPs. This observation was further supported by the fact that the higher the expression variation of a RBP in breast cancer patients, the higher the number of protein–protein interactions. This indicates that fluctuating RBPs whose expression is not tightly controlled (with differing fold changes in expression levels across patients) might be involved in more promiscuous (non-specific) protein interactions thereby leading to variable RNA off-targets at the post-transcriptional level.

To further determine the prognostic impact in breast cancer patients we ranked the SUR and non-SUR RBPs based on path length. The two RBP groups had different distributions. We found that as the mean path lengths between SUR RBPs increased their contribution to prognostic impact increased, suggesting that SUR RBPs with higher network distances with respect to other SUR RBPs, are more likely to contribute independently to survival as they might influence a larger fraction of the dysregulated network of SUR RBPs. In contrast, when a non-SUR RBP had a shorter path to a SUR RBP, there was a significant prognostic impact. This suggests that they are closer to the actual contributors of pathogenesis at the post-transcriptional level; however, the longer the path lengths, the weaker the prognosis. To gain further insight into the contribution of these subsets of RBPs in the development of and survival with cancer, we compared the overall significance of the Kaplan–Meier *P* values (-log[*P*]) for groups of RBPs classified by their level of dysregulation (SUR vs non-SUR). This analysis revealed no significant differences between groups of SUR and non-SUR RBPs in terms of their prognosis for survival. However, we found that, in general, the higher the expression variation across patients, the lower the prognostic impact of the protein. Our results suggest that RBPs from our signature set with lower variation in expression levels across patients might be good starting points for studying the effect of RBPs in cancer pathogenesis since SUR RBPs with large expression fold changes might be downstream or there might be indirect effects (Additional file 8: Figure S6). Additionally, common factors that are dysfunctional along the shortest paths in the protein interaction networks of SUR RBPs could also provide clues for potential drug targets as they can act as regulators for rewiring the post-translational landscape of RBPs thereby affecting RNP complex formation. With increasing efforts to uncover the binding sites of RBPs in higher eukaryotes using a variety of high-throughput approaches [69,70], it should also become possible in the near future to study the differences in the target RNA pools between healthy and cancer genomes for several of these SUR RBPs. This would provide a global picture of the affected post-transcriptional regulatory networks. The global integration of networks governed by post-transcriptional players like miRNAs and RBPs together with signaling networks can provide a comprehensive picture of the cause of the dysregulation in these RBPs, which can be used to tease apart the contributions of local malfunctions and those due to an upstream or downstream effect in the cellular networks.

## Materials and methods

### Data for healthy expression of RNA-binding proteins in 16 human tissues

Our general workflow is illustrated in Figure 1. RNA-seq data for 16 different human tissues from ArrayExpress [71] (Accession no. E-MTAB-513), which is part of the Human BodyMap (HBM) 2.0 project [18,22], was obtained for expression profiling. This data represents the healthy RNA transcript levels of male and female individuals aged 19 to 86, for 16 tissues: adipose, adrenal, brain, breast, colon, heart, kidney, liver, lung, lymph node, ovary, prostate, skeletal muscle, testes, thyroid and white blood cells. Expression data from the HBM project was quantified per transcript using the current annotations of the human genome from the Ensembl. This is available as reads per kilobase per millions of reads (RPKM) for each sample and hence can be compared across and within tissues. Therefore, each of the 16 tissues has a single RPKM value for the expression level of each transcript. A total of 850 genes experimentally characterized as RBPs in the human genome were obtained from a previous publication [17] and 4,647 transcripts associated with these RBPs were identified within the HBM set. The remaining set of 102,462 transcripts were classified as non-RBPs in this study. To examine the other regulatory factors in humans we obtained a set of 9,440 long non-coding RNAs (lncRNAs) from a Gencode study [18,72], 529 microRNAs (miRNAs) from miRBase [73] and 1,231 transcription factors (TFs) from the DBD database [74] (Additional file 2: Table S1). For each of the 16 tissues we compared the distribution of the RPKM values for transcripts associated with RBPs and non-RBPs, as well as the distribution of expression levels of transcripts associated with RBPs with other regulatory factors to study their relative effect on regulatory control at the tissue level.

### Data for cancer expression of RNA-binding proteins for nine cancers in humans

The cancer expression data was downloaded from TCGA [19]. TCGA provides multi-level data (clinical, genome sequencing, microarray, RNA sequencing and so on) procured from a number of institutions, from a variety of patients, for over 25 cancers. In this study, we collected RNAseq V2.0 data for 2,876 patients spanning nine cancers analogous to eight of our tissues in the HBM dataset: breast (850 patients), brain (175 patients), colon (193 patients), kidney (481 patients), liver (35 patients), two for lung (356 and 260 patients), prostate (141 patients), and thyroid (385 patients). TCGA accession numbers for the patient samples used in this study are available in Additional file 9: Table S3. For each cancer we collected the expression levels for each gene for all patients and determined a median representative level and MAD. This defines the genes’ RNA expression levels and variability in the relevant cancer state. Likewise, cancer expression and variation were determined for the group of non-RBP genes from HBM as a complementary group for later network, interaction, and expression analyses. Hierarchical clustering of RBP expression for these nine cancers was performed in R, to determine if similar cancers and tissues group together (Additional file 3: Figure S2). Clustering results verified that the collected and amalgamated data are an accurate representation of their anatomical origin, and can be utilized to draw further conclusions.

### Profiling for dysregulation of RNA-binding proteins and identification of strongly upregulated RNA-binding proteins across human cancers

For each gene identified as an RBP, we calculated a median expression level of its transcript products in the HBM data when there were multiple protein coding transcripts. To determine the extent of dysregulation in RBPs across cancers, we calculated for each cancer the log-ratio of the median expression in the cancer state over its expression in the associated healthy state. This allowed us to determine for the nine cancers if a particular gene annotated as an RBP is upregulated, downregulated or does not change in expression level in cancer states. Based on this analysis, if an RBP has a log-ratio of expression level greater than 9 across six or more of the studied cancers, we classified it as being SUR. Otherwise, it was categorized as non-SUR. We focused mainly on defining characteristics unique to these SUR RBPs that differentiate them from other RBPs and non-RBPs. SUR genes as defined here were also observed in non-RBPs and a hypergeometric test was performed to examine potential differences in the proportionality of SUR RBPs and non-SUR RBPs between the two functional classes. The genes associated with RBPs and non-RBPs were also classified by their level of expression variability in a cancer, measured as the MAD value of the fold change in expression for the profiled patients for the cancer. If a gene’s variability within a cancer was above the 75th percentile, it was considered highly variable, below the 25th percentile it was considered least variable and the remainder were considered moderately variable.

### Network and interaction properties of dysregulated RNA-binding proteins in human cancers

The most recent BioGRID [66] protein–protein interaction (PPI) information (version 3.2.97) was downloaded and used to construct an undirected network of interactions documented in humans. These interactions were used to determine if there were any differences in network properties between the two classifications of dysregulated RBPs, that is, SUR and non-SUR RBPs. This allowed the determination of the potential importance of the classifications for these RBPs. For example, if an SUR RBP forms a hub, it could cause patterns of dysregulation in other, associated interactors. We compared network centrality measures such as degree, closeness and betweenness as well as clustering coefficients and shortest paths between nodes, for different RBP classes utilizing the R package igraph [75]. For shortest paths, we calculated the mean shortest paths for a SUR RBP to other SUR RBPs and SUR RBPs to non-SUR RBPs. We also obtained the overall average path length between each RBP/non-RBP and SUR RBP/non-SUR RBP combination.

Manually curated experimentally characterized human protein complex data was obtained from CORUM [76], to determine the general promiscuity of RBPs in forming complexes. Then 5,217 protein complexes were mapped to the RBPs. We calculated for SUR RBPs and non-SUR RBPs the frequency of membership in CORUM complexes, as well as the mean complex size. This information together with the log-ratios of expression levels between healthy and cancer states in the tissues, allowed us to address whether SUR RBPs are enriched in protein complexes and/or occur in larger or smaller complexes. This analysis also allowed us to test the relation between the extent of an RBP’s dysregulation in the context of its membership.

### Determination of prognostic impact of RNA-binding proteins for breast cancer

A gene’s prognostic impact is the gene’s ability to impact positively or negatively patient survival. The prognostic impact for each gene was determined using data from the Kaplan–Meier (KM)-Plotter [68], which was determined from microarray experiments for over 20,000 genes for 1,800 breast cancer patients. For each gene in the RBP and non-RBP groups, we further categorized them as SUR or non-SUR and high or low variability in expression. We compared the significance [-log(KM-plotter *P*)] of the prognostic impacts within and between these groups.

Based on the network analyses, the genes were ranked in descending order based on their mean path lengths to the classification of dysregulated genes (SUR vs non-SUR). Path length calculations were determined from a distance matrix generated by the network analysis. From the ranked list of genes we selected five genes with the shortest and longest mean path lengths, and took a random sample of five genes with intermediate mean path lengths. This provided information on the prognostic impact associated with increased gene expression.

## Abbreviations

CLIP: cross-linking and immunoprecipitation; HBM: Human BodyMap; KM: Kaplan–Meier; Kolmogorov–Smirnov test: KS test; lncRNA: long non-coding RNA; MAD: median absolute deviation; miRNA: microRNA; PAR-CLIP: photoactivatable-ribonucleoside-enhanced CLIP; PHD: plant homeodomain; PPI: protein–protein interaction; RBP: RNA-binding protein; RNA-seq: RNA sequencing; RNP: ribonucleoprotein; RPKM: reads per kilobase per millions of reads; SUR: strongly upregulated; TCGA: the Cancer Genome Atlas; TF: transcription factor; TNF: tumor necrosis factor; KS test: Kolmogorov–Smirnov test.

## Competing interests

The authors declare that they have no competing interests.

## Authors' contributions

BK performed the experiments. SCJ conceived and designed the experiments. BK and SCJ analyzed the data, contributed reagents, materials and analysis tools and drafted the manuscript. Both authors read and approved the final manuscript.

## Supplementary Material

Additional file 1: Figure S1 Expression levels of RNA-binding proteins (RBPs), non-RBPs, lncRNAs, miRNAs and transcription factors (TFs) for 16 human tissues. Each of the 16 plots illustrates the significant differences in expression levels of RBPs (*P* < 2 × 10^-16^, Wilcox test) for adipose, adrenal, brain, breast, colon, heart, kidney, liver, lung, lymph node, ovary, prostate, skeletal muscle, testes, thyroid and white blood cell tissues, compared to the other regulatory factors. The *x*-axis is the category of the observed factor and the *y*-axis is the expression level. Click here for file

Additional file 2: Table S1 Expression values for transcripts from HBM data for RBPs, lncRNAs, miRNAs and transcription factors. The statistical analysis compares the expression levels of RBPs and TFs for various tissues. Included are log-fold changes in expression levels for RBPs across nine cancers and *P* values from *t*-tests comparing tumor and tumor-matched normal cancer samples. Additionally the statistical significance for interaction and expression/variation classifications are shown. Click here for file

Additional file 3: Figure S2 Correlation matrix of overall log-ratio expression of RBPs across nine cancers. The matrix shows the clustering of similar tissue sites and similar cancer types. Click here for file

Additional file 4: Table S2 Strongly upregulated RNA-binding proteins (SUR RBPs), including functional description, currently annotated disease associations and additional database identifiers. Click here for file

Additional file 5: Figure S3 Comparison of normalized network metrics (closeness, betweenness and degree) between strongly upregulated (SUR) and non-strongly upregulated (non-SUR) RNA-binding proteins. The median values for each property are the same and there are no significant differences (*P* > 0.05, Wilcox test). Click here for file

Additional file 6: Figure S4 CORUM complex membership and complex size distribution for strongly upregulated (SUR) and non-strongly upregulated (non-SUR) RNA-binding proteins. There were no significant differences between the two groups (*P* > 0.05, Wilcox test). Click here for file

Additional file 7: Figure S5 CORUM complex membership and complex size distribution vs expression for strongly upregulated (SUR) and non-strongly upregulated (non-SUR) RNA-binding proteins. No trends were observed when comparing the CORUM characteristics with expression. Click here for file

Additional file 8: Figure S6 Heat map showing the variation in expression level measured as median absolute deviation (MAD) values for SUR RNA-binding proteins for nine types of cancer. Click here for file

Additional file 9: Table S3 Accession numbers of TCGA patient samples for all nine cancers analyzed in this study. Click here for file
