# Supplementary figures and images for: Dissecting the expression landscape of RNA-binding proteins in human cancers
Source: Genome Biol. 2014 Jan 10;15(1):R14. doi: 10.1186/gb-2014-15-1-r14 (PMC4053825; doi:10.1186/gb-2014-15-1-r14)

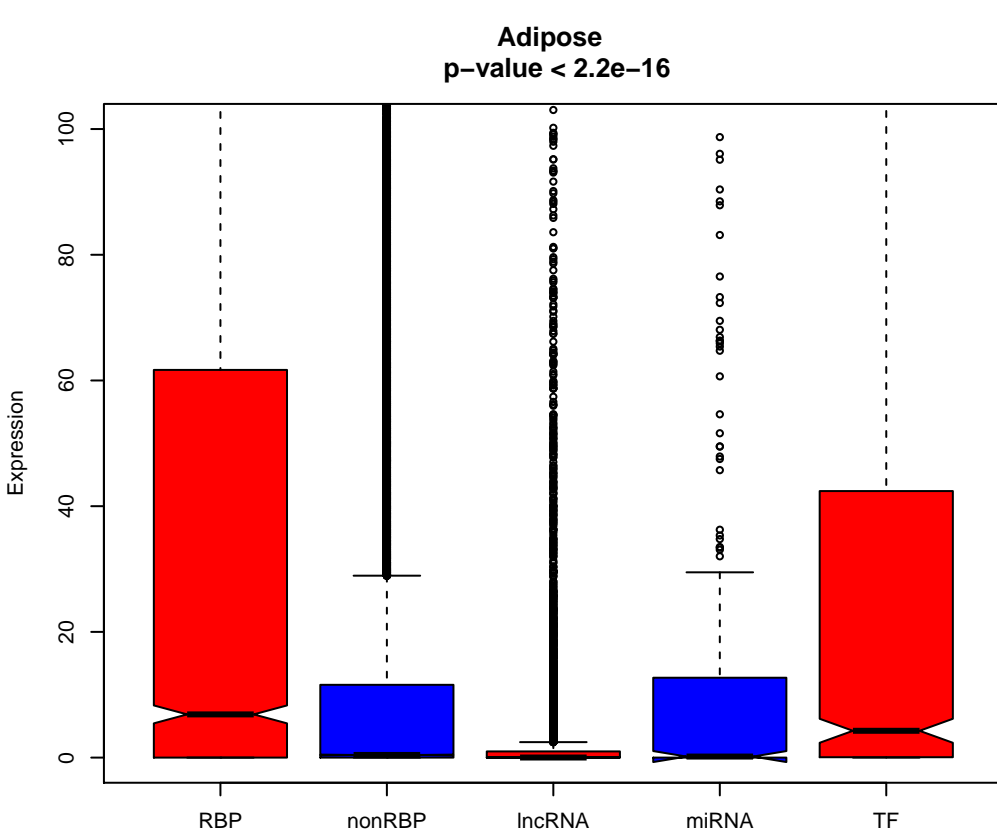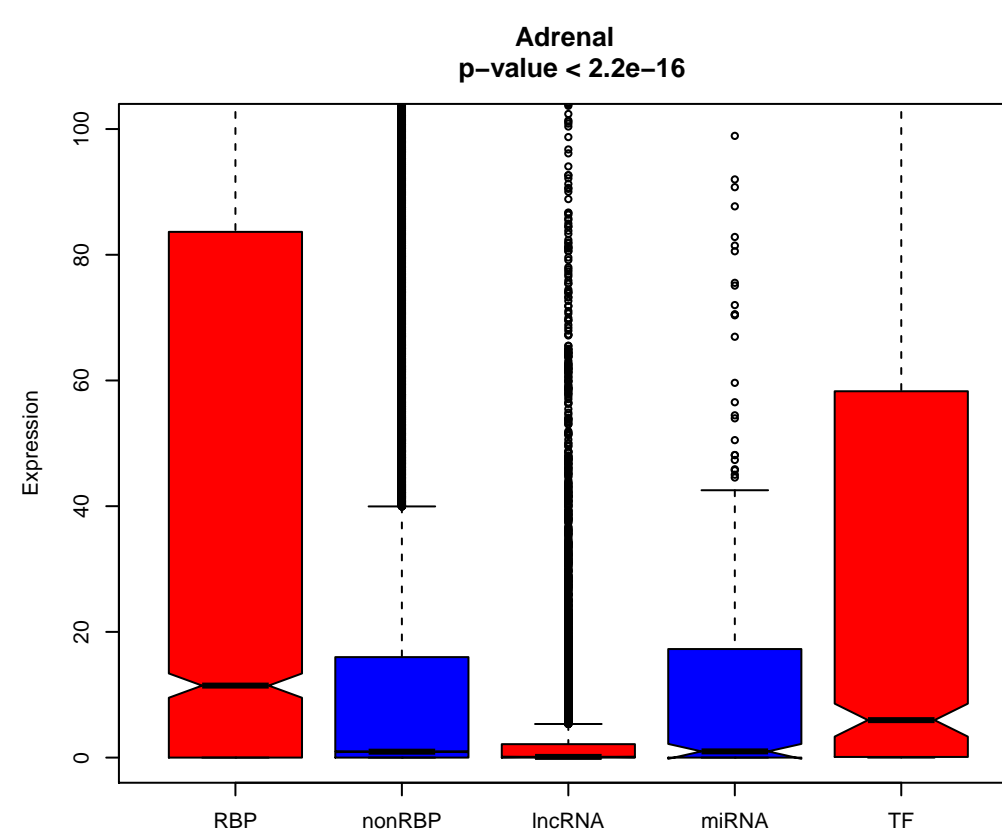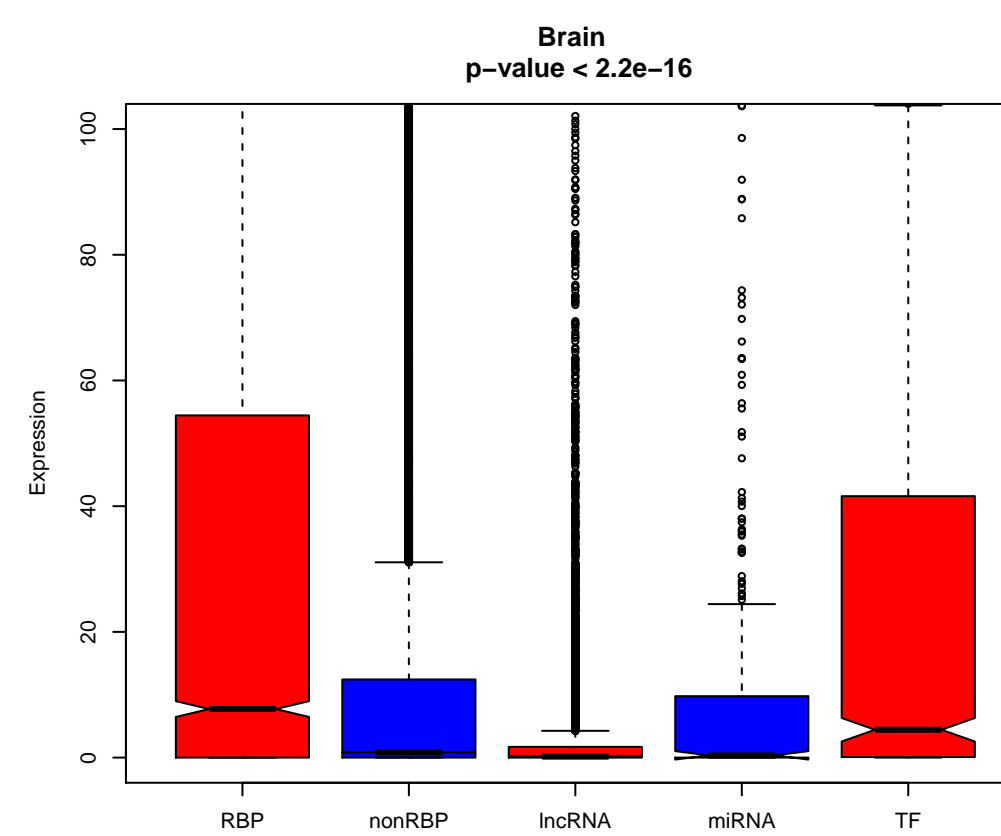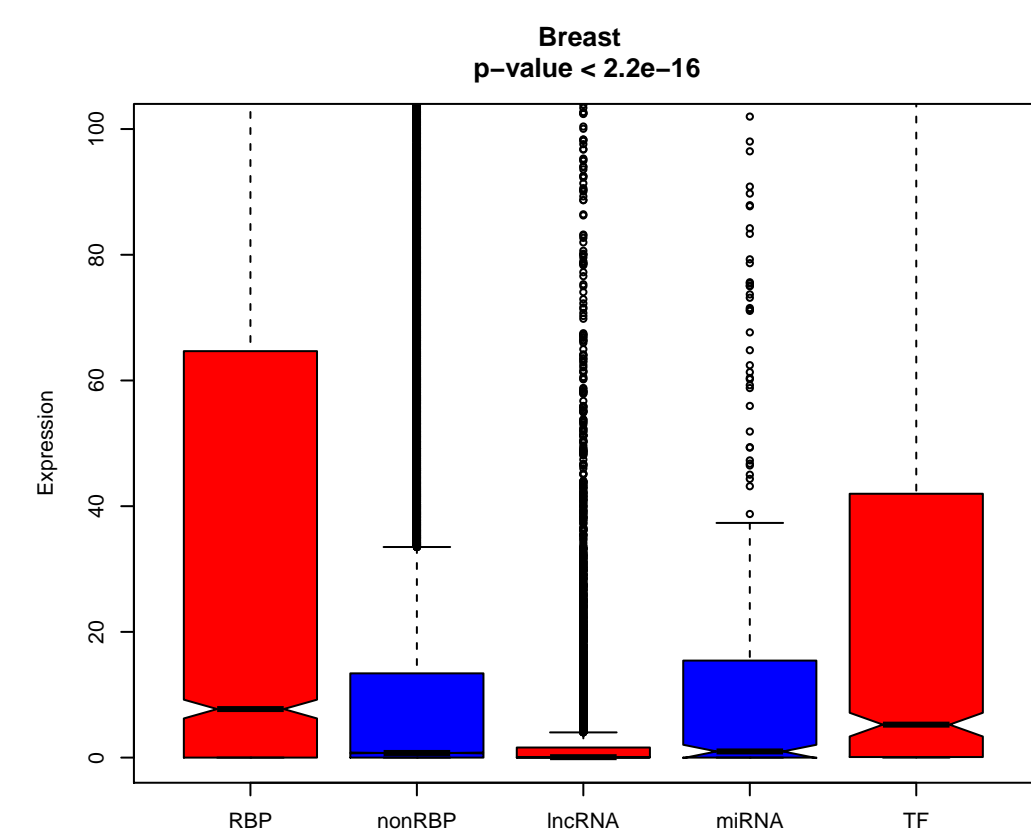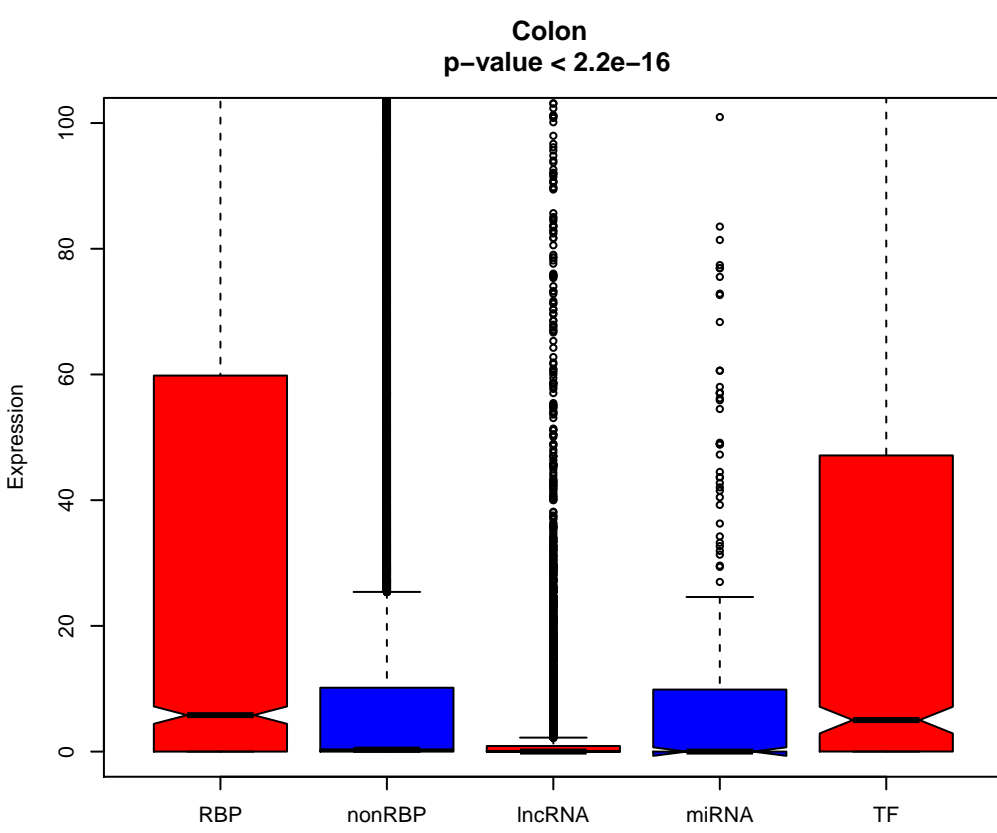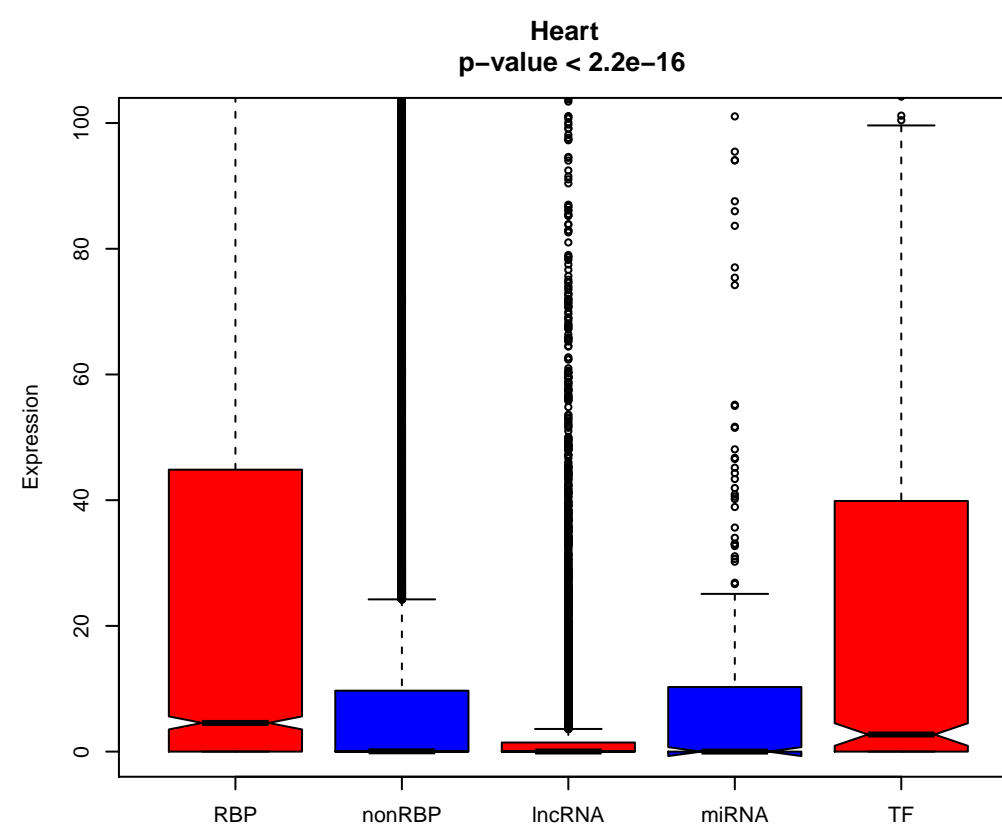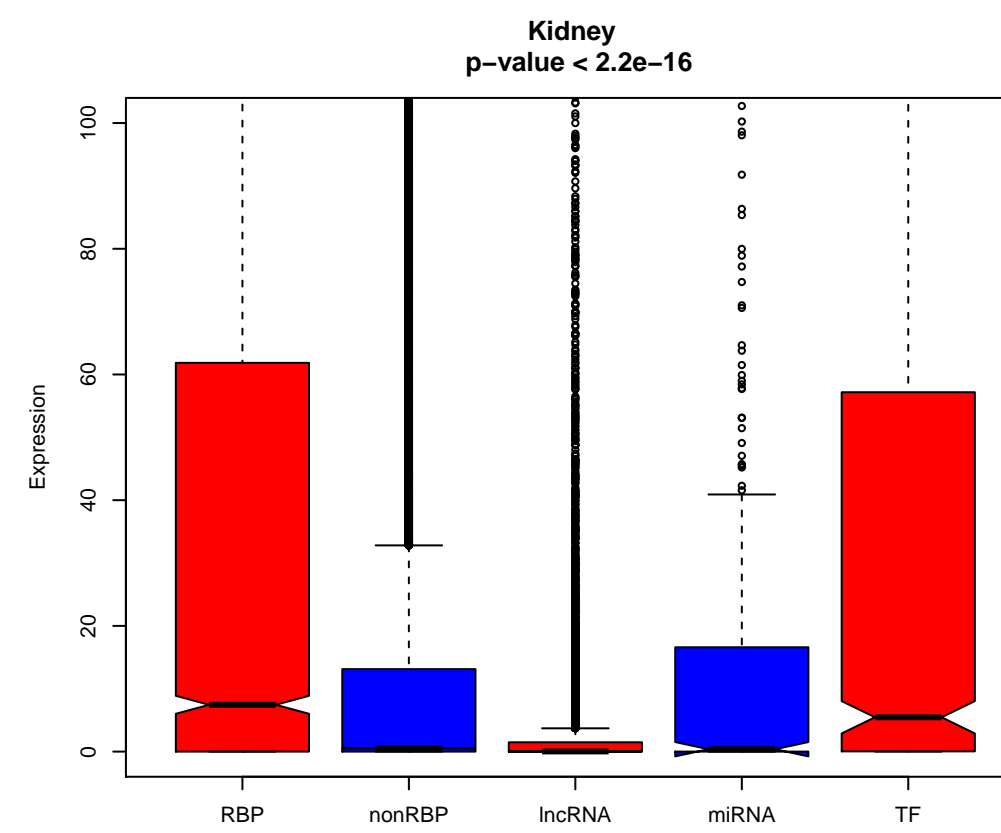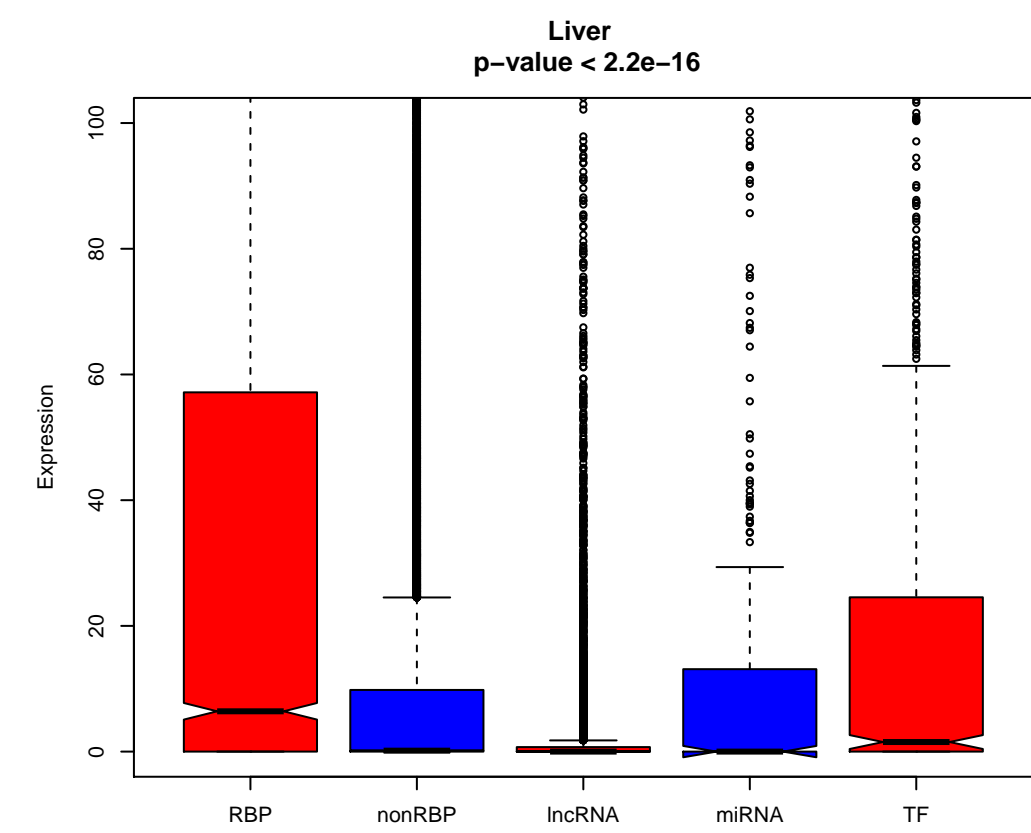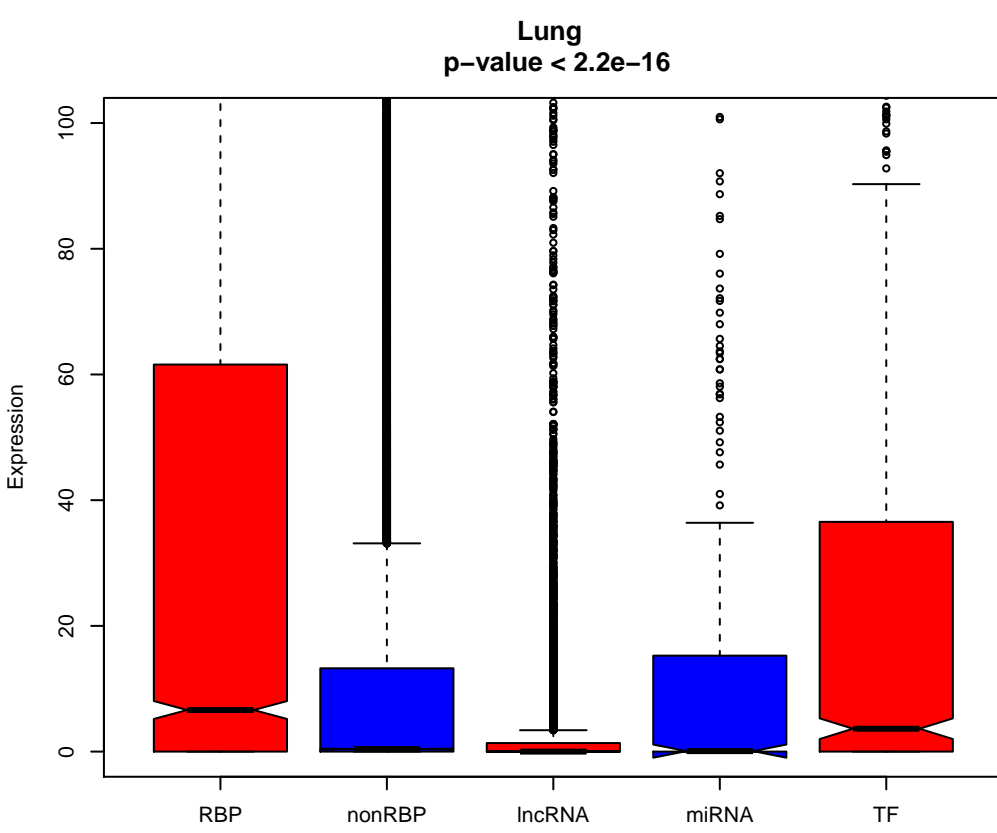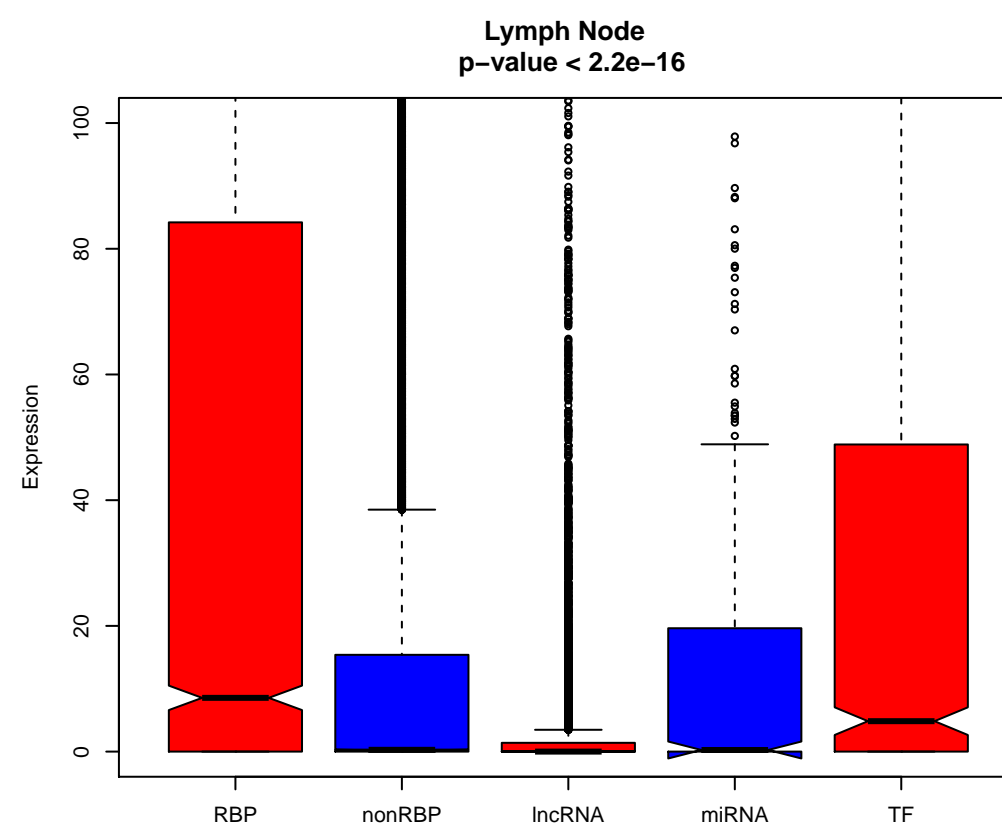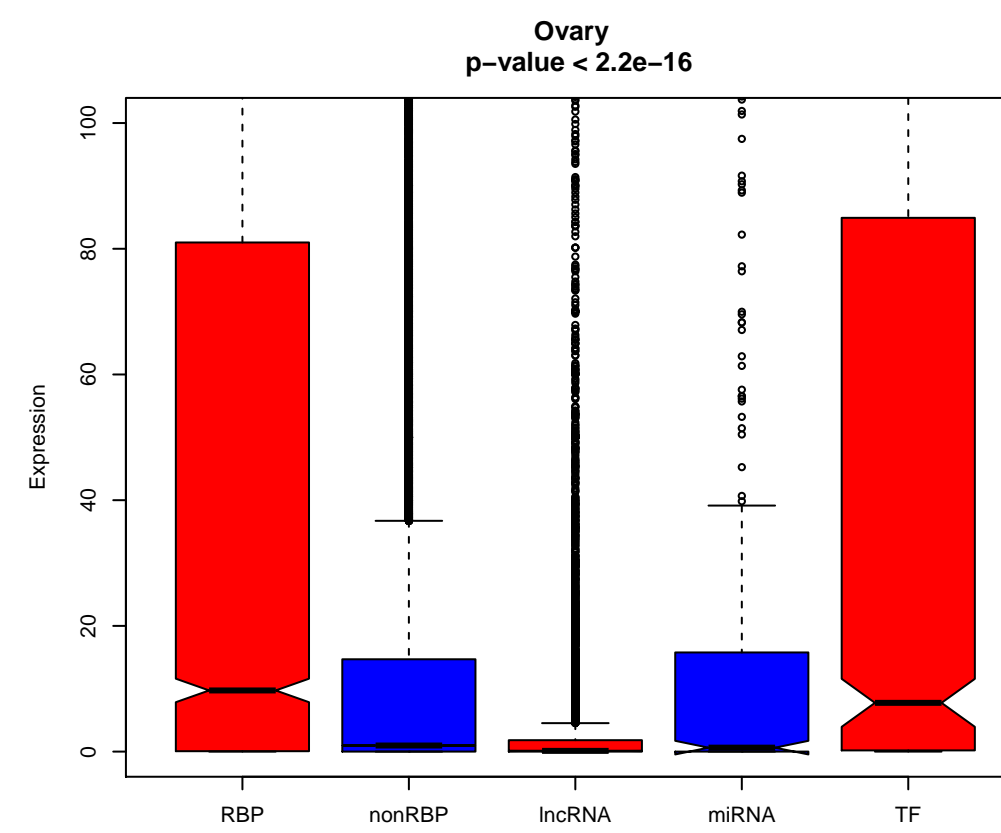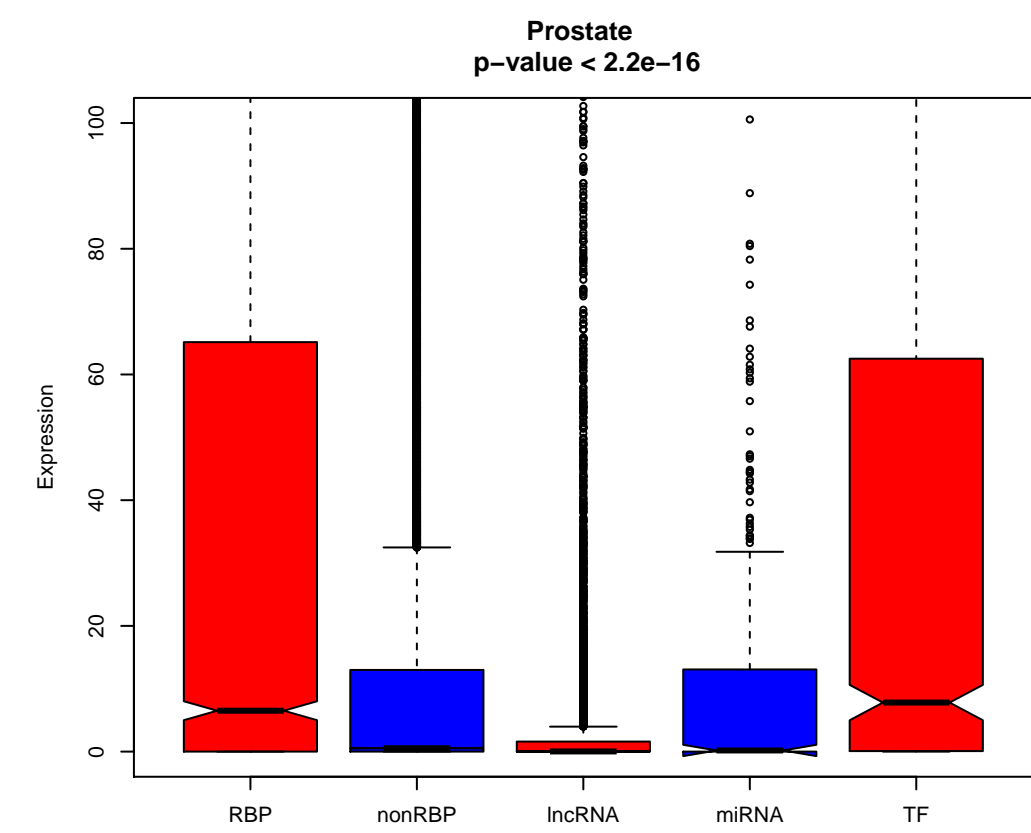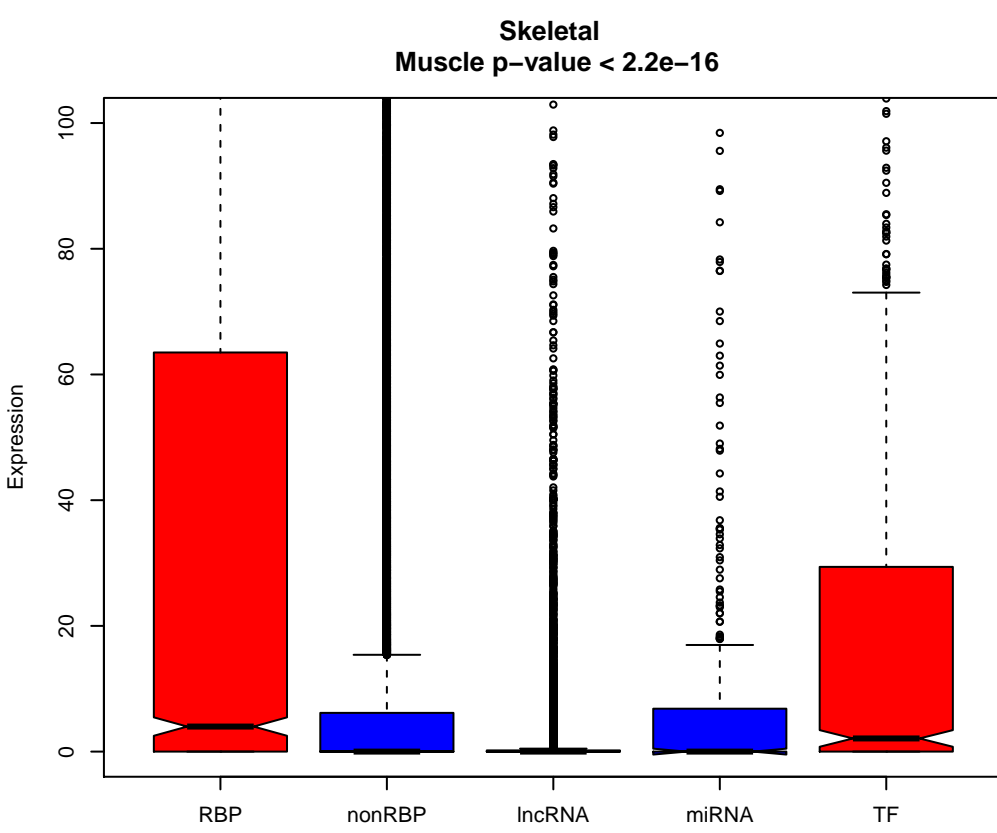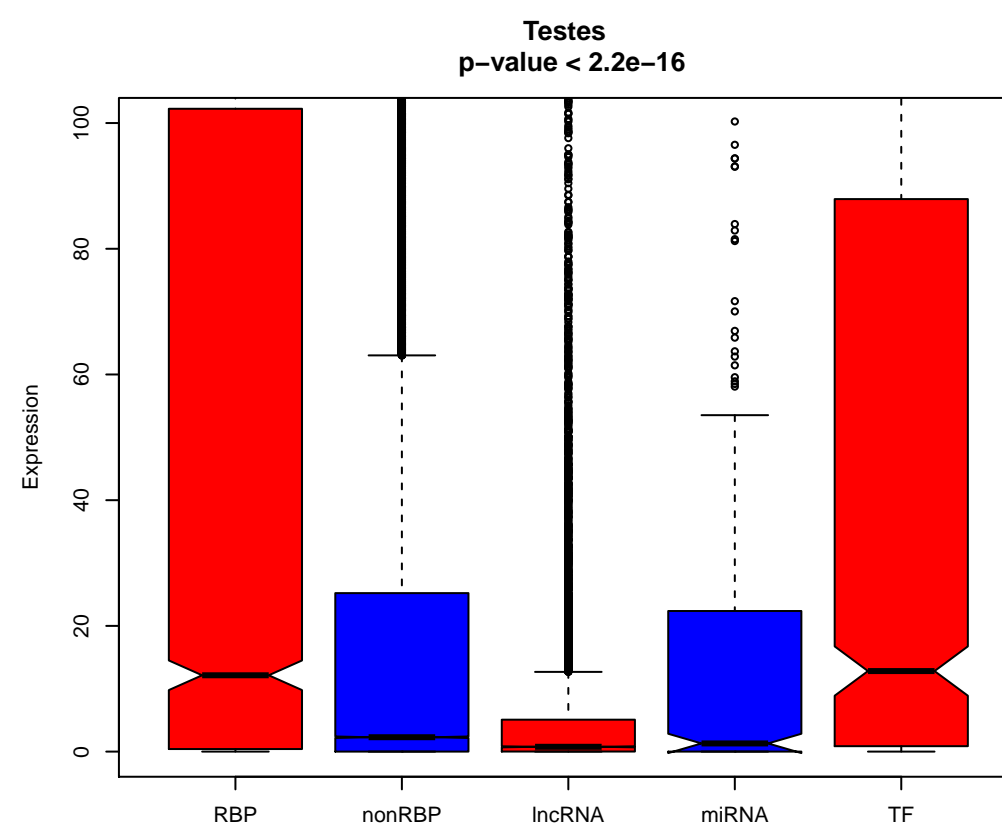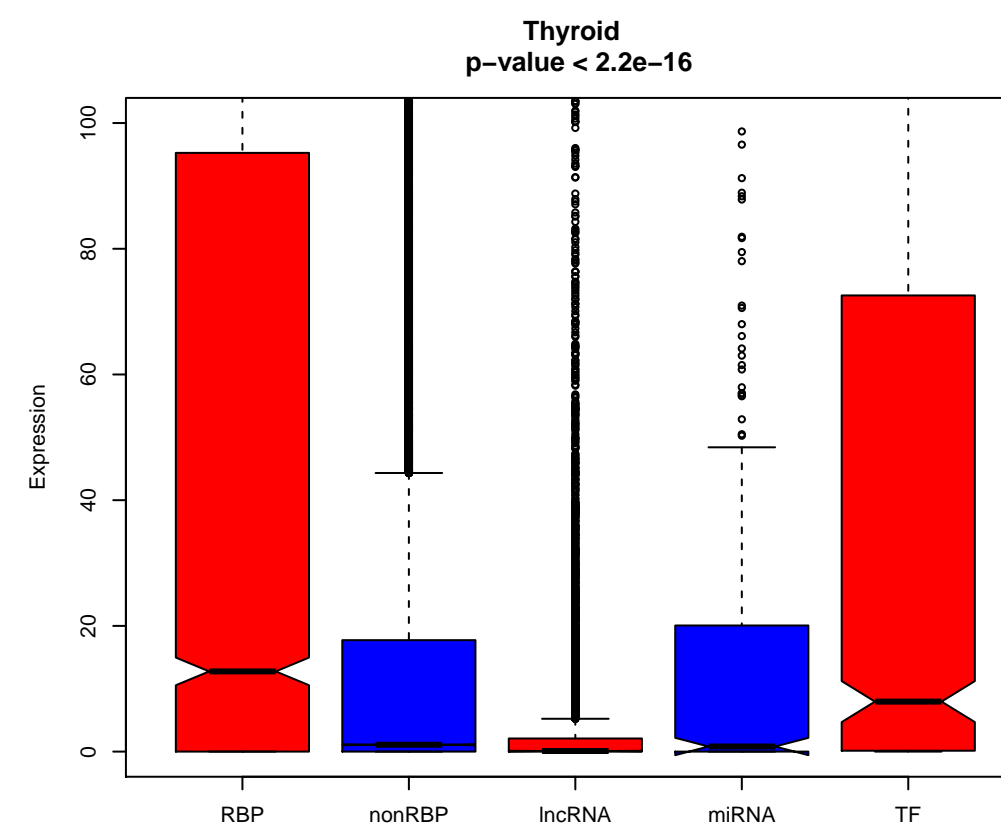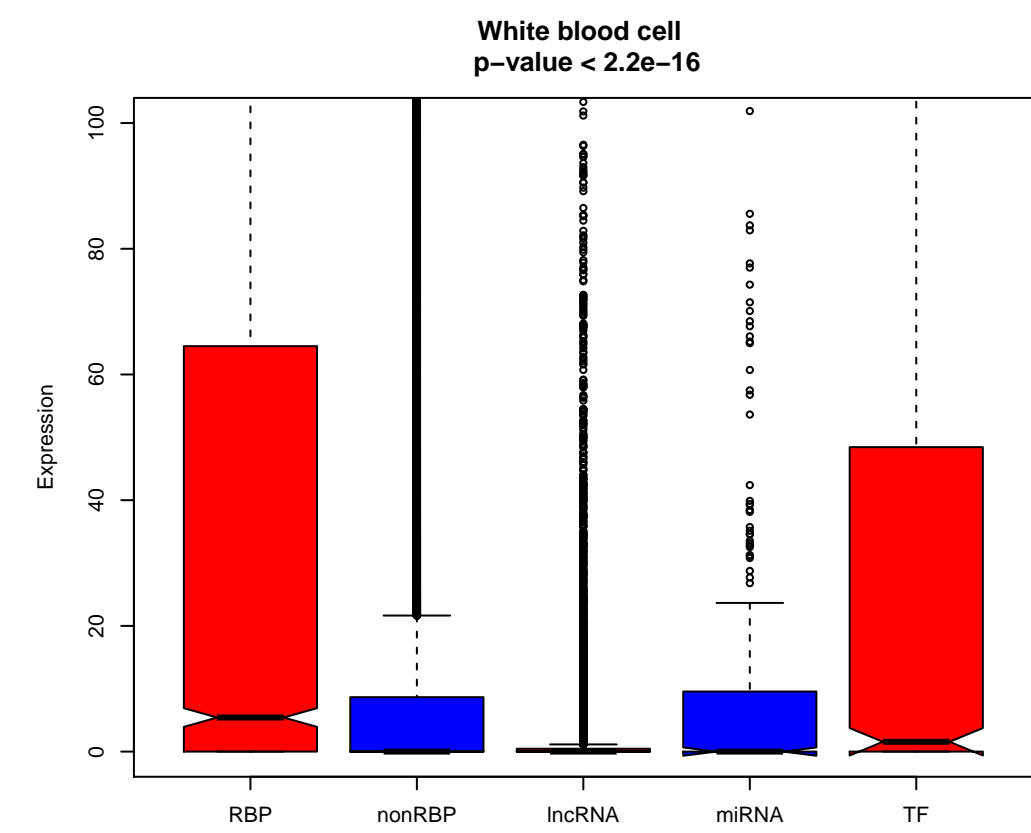

Supplement: Additional file 1: Figure S1 — Expression levels of RNA-binding proteins (RBPs), non-RBPs, lncRNAs, miRNAs and transcription factors (TFs) for 16 human tissues. Each of the 16 plots illustrates the significant differences in expression levels of RBPs (P < 2 × 10^-16^, Wilcox test) for adipose, adrenal, brain, breast, colon, heart, kidney, liver, lung, lymph node, ovary, prostate, skeletal muscle, testes, thyroid and white blood cell tissues, compared to the other regulatory factors. The x-axis is the category of the observed factor and the y-axis is the expression level. [file gb-2014-15-1-r14-S1.pdf]

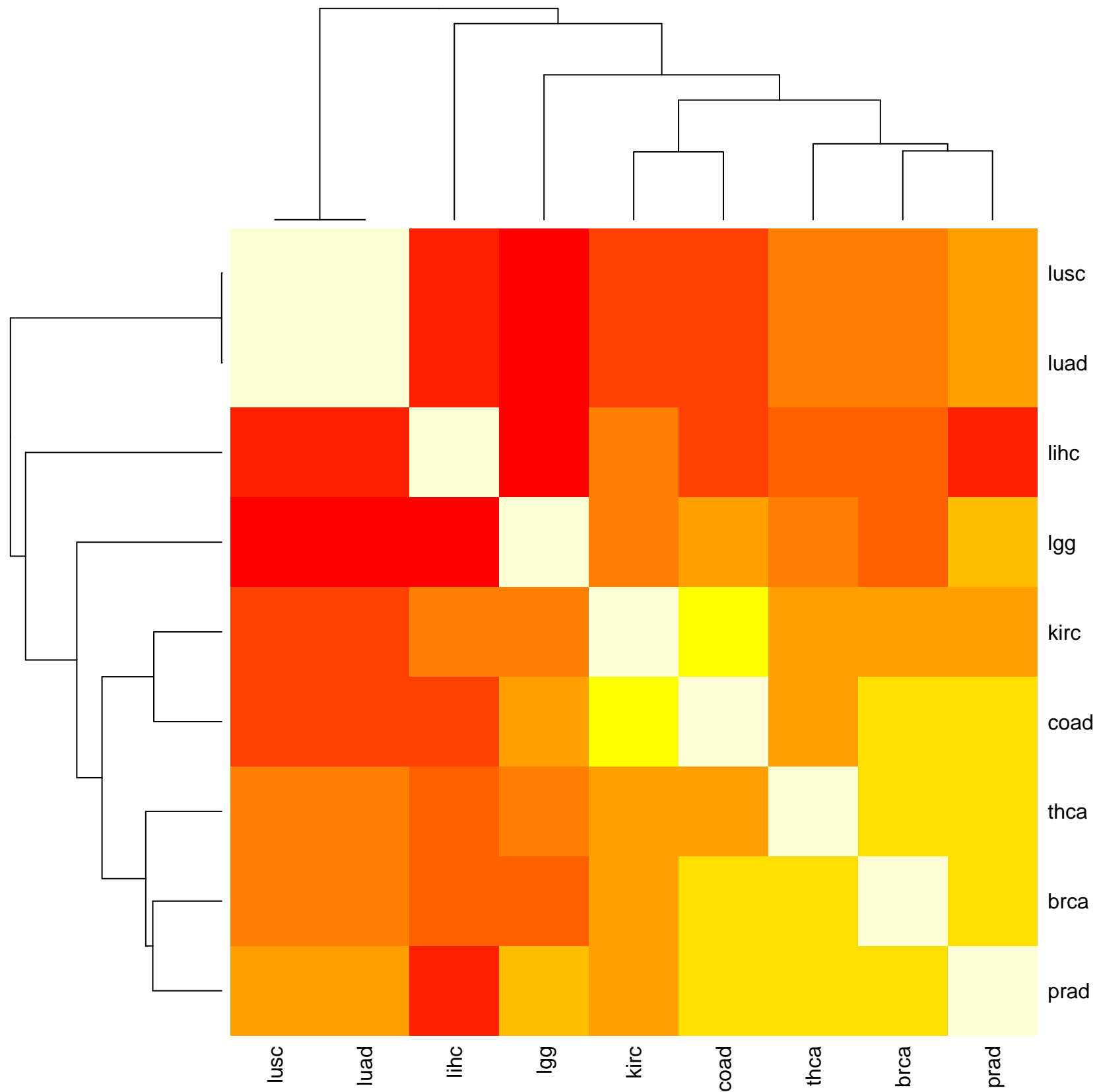

Supplement: Additional file 3: Figure S2 — Correlation matrix of overall log-ratio expression of RBPs across nine cancers. The matrix shows the clustering of similar tissue sites and similar cancer types. [file gb-2014-15-1-r14-S3.pdf]

A Shortest path distributions

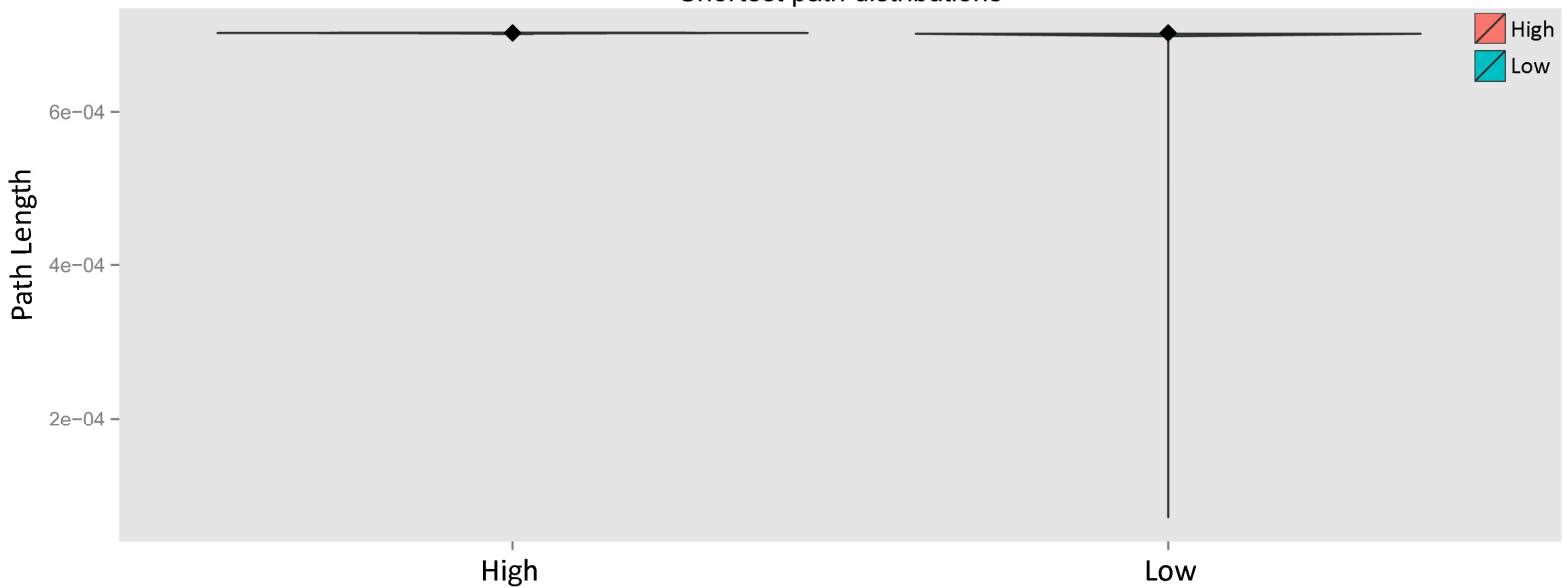

B Normalized degree distribution

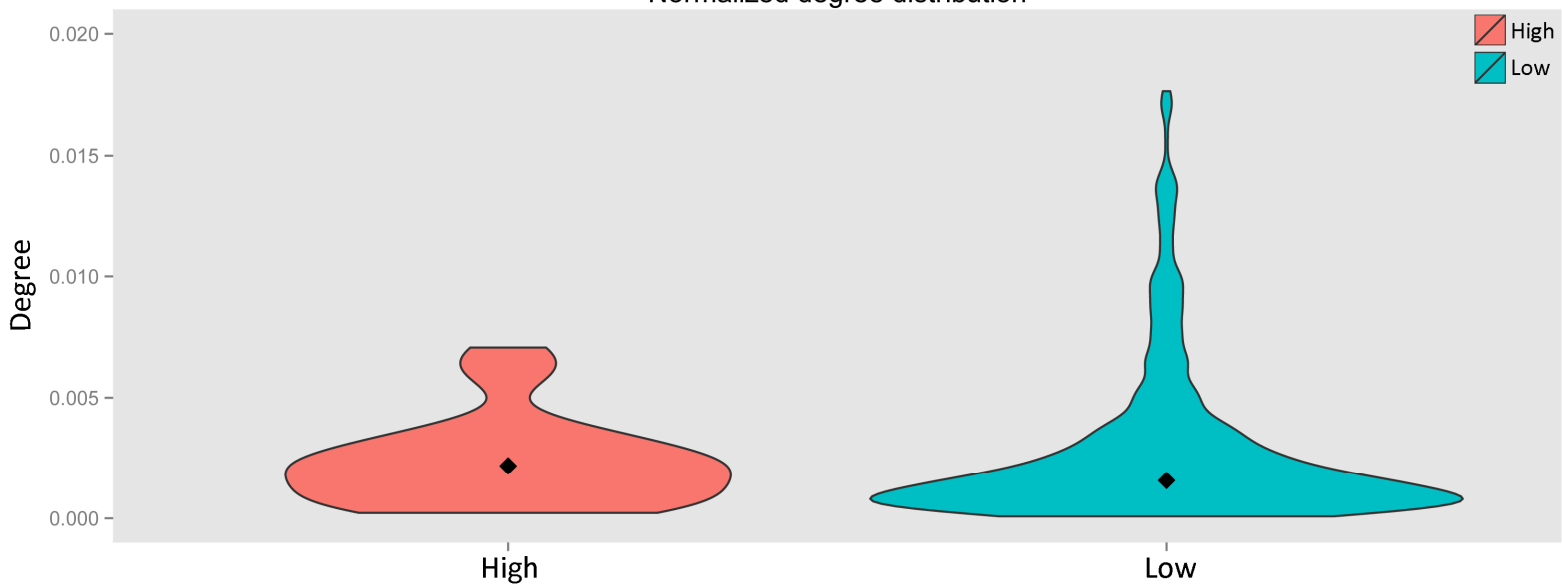

C Normalized Betweenness

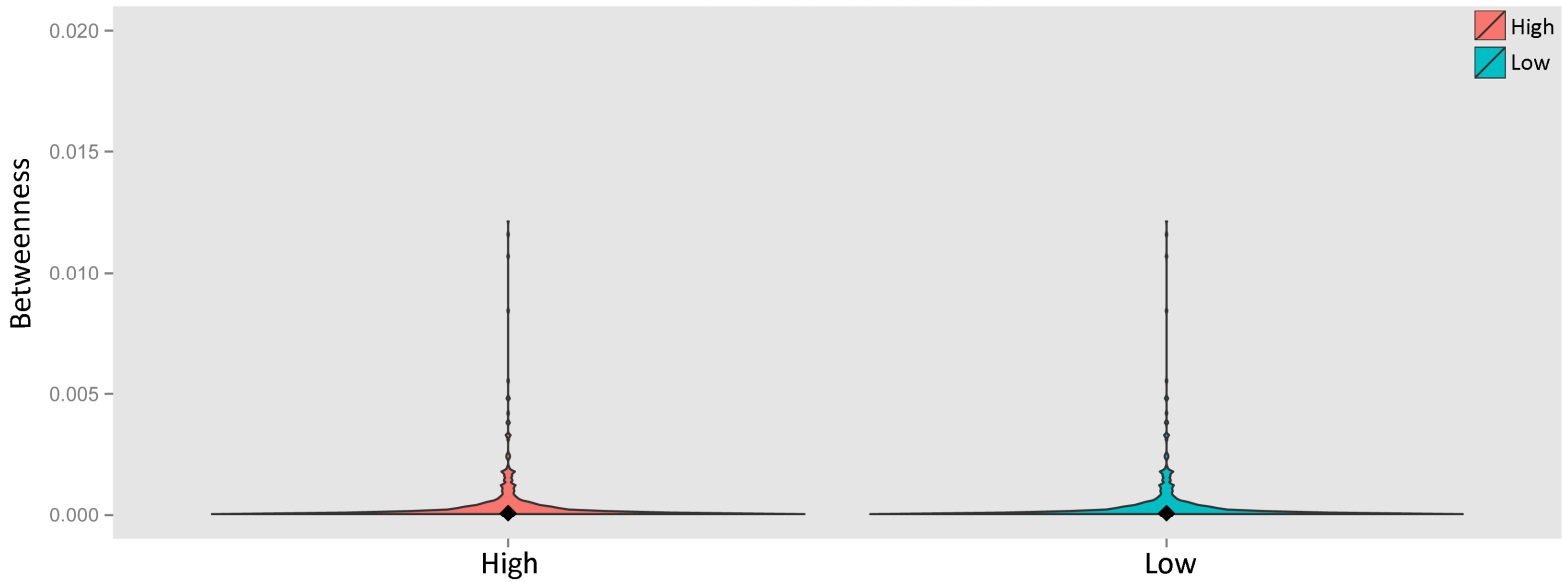

Supplement: Additional file 5: Figure S3 — Comparison of normalized network metrics (closeness, betweenness and degree) between strongly upregulated (SUR) and non-strongly upregulated (non-SUR) RNA-binding proteins. The median values for each property are the same and there are no significant differences (P > 0.05, Wilcox test). [file gb-2014-15-1-r14-S5.pdf]

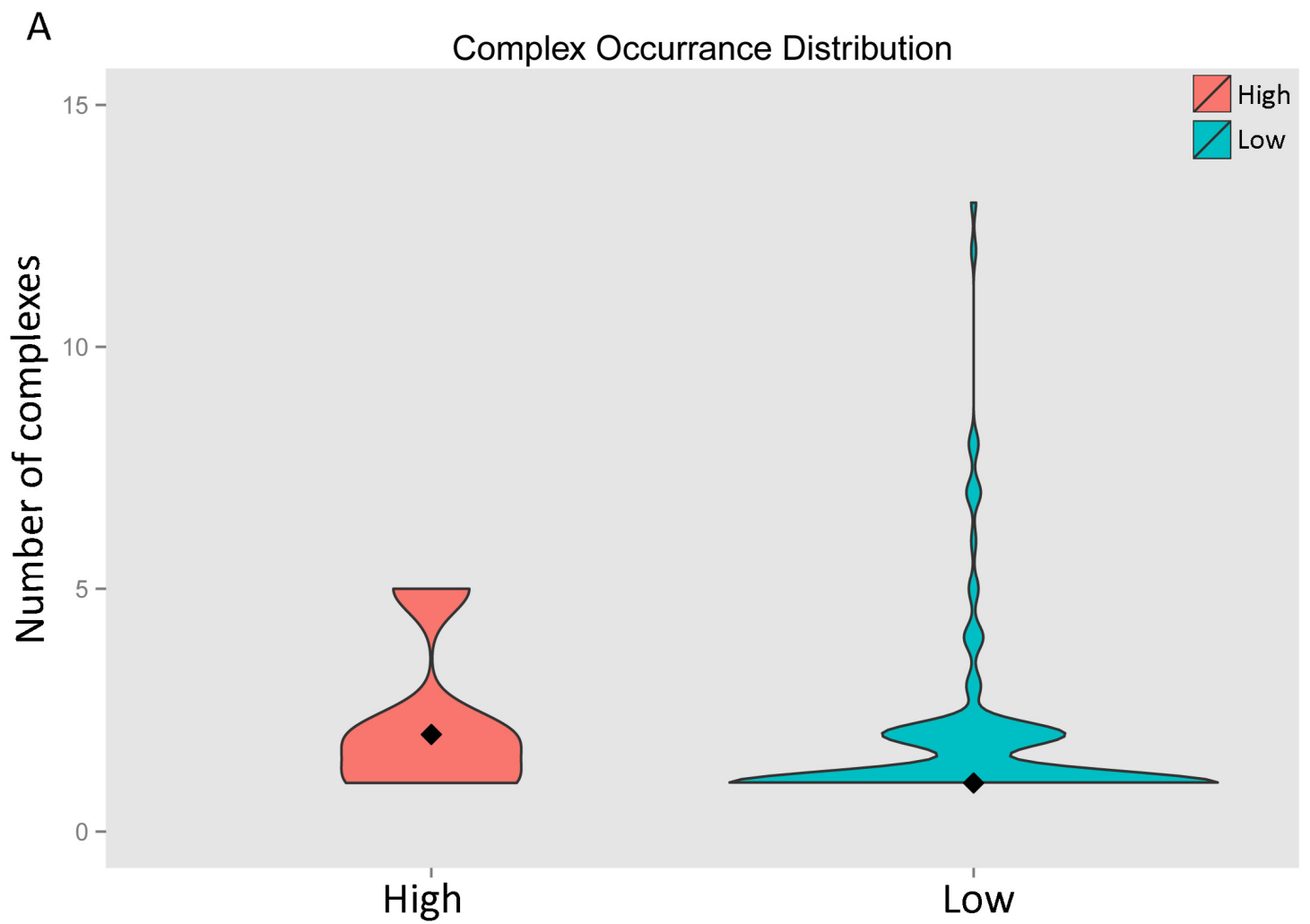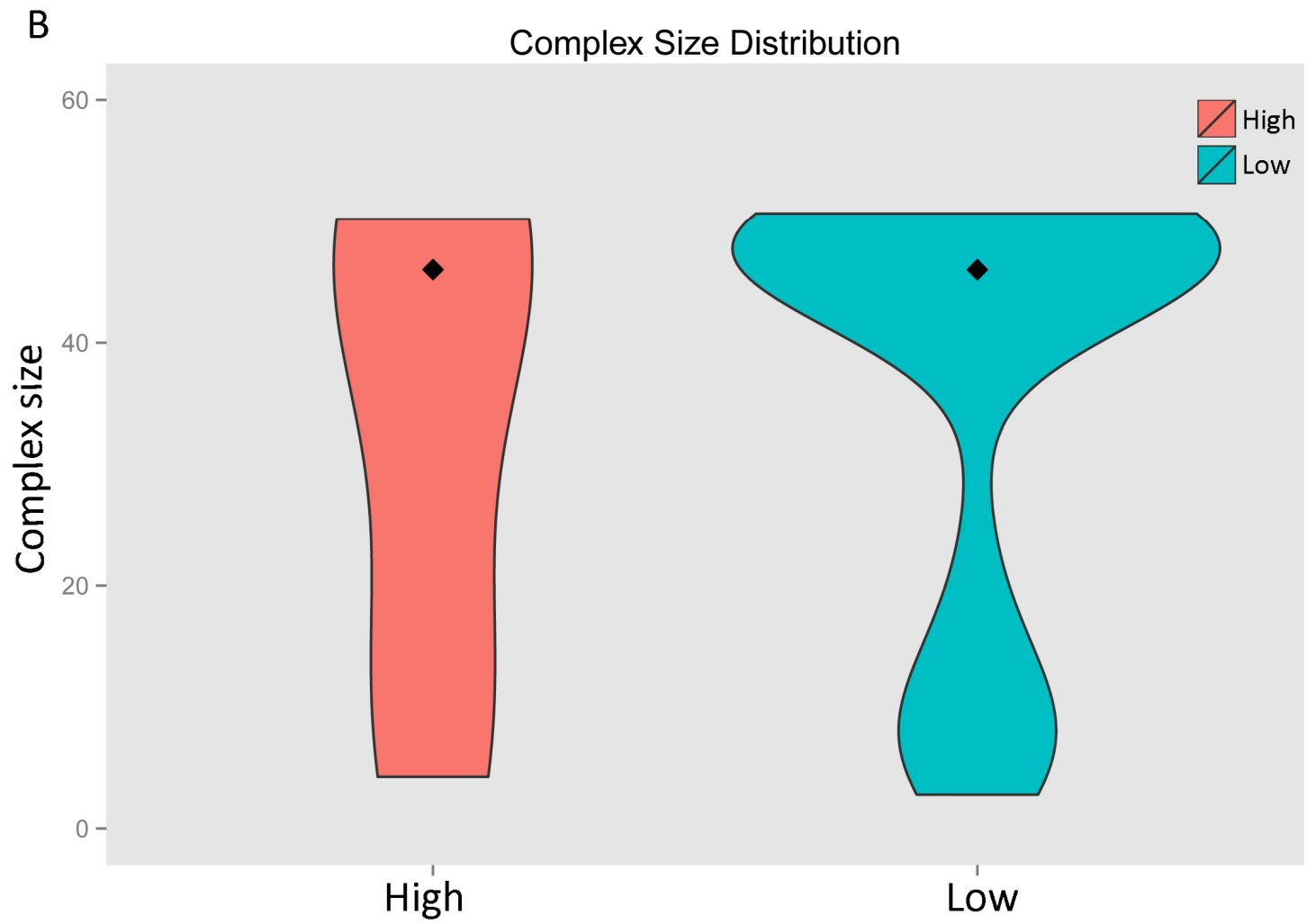

Supplement: Additional file 6: Figure S4 — CORUM complex membership and complex size distribution for strongly upregulated (SUR) and non-strongly upregulated (non-SUR) RNA-binding proteins. There were no significant differences between the two groups (P > 0.05, Wilcox test). [file gb-2014-15-1-r14-S6.pdf]

A

Expression versus Complex count

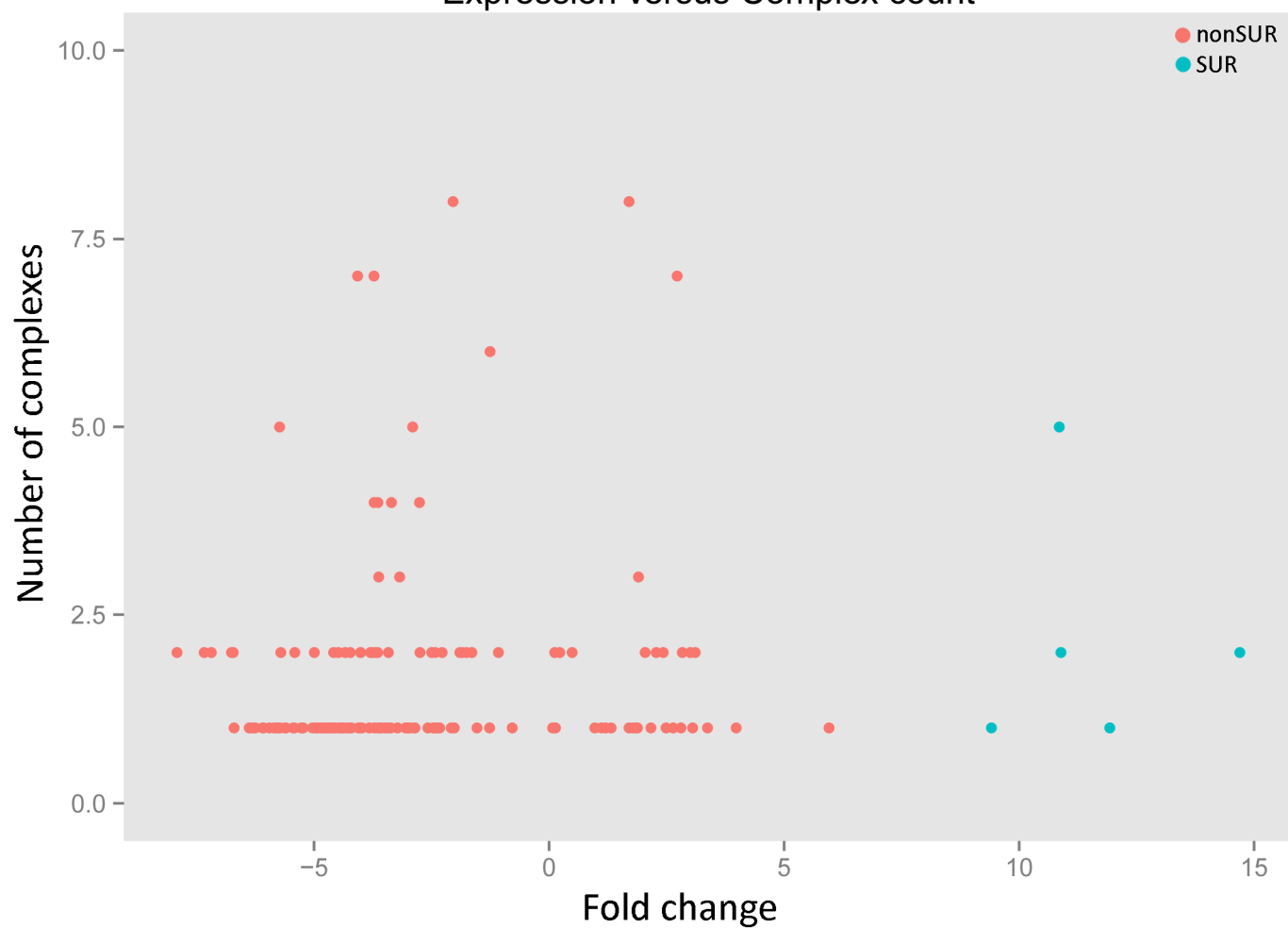

B

Expression versus Complex size

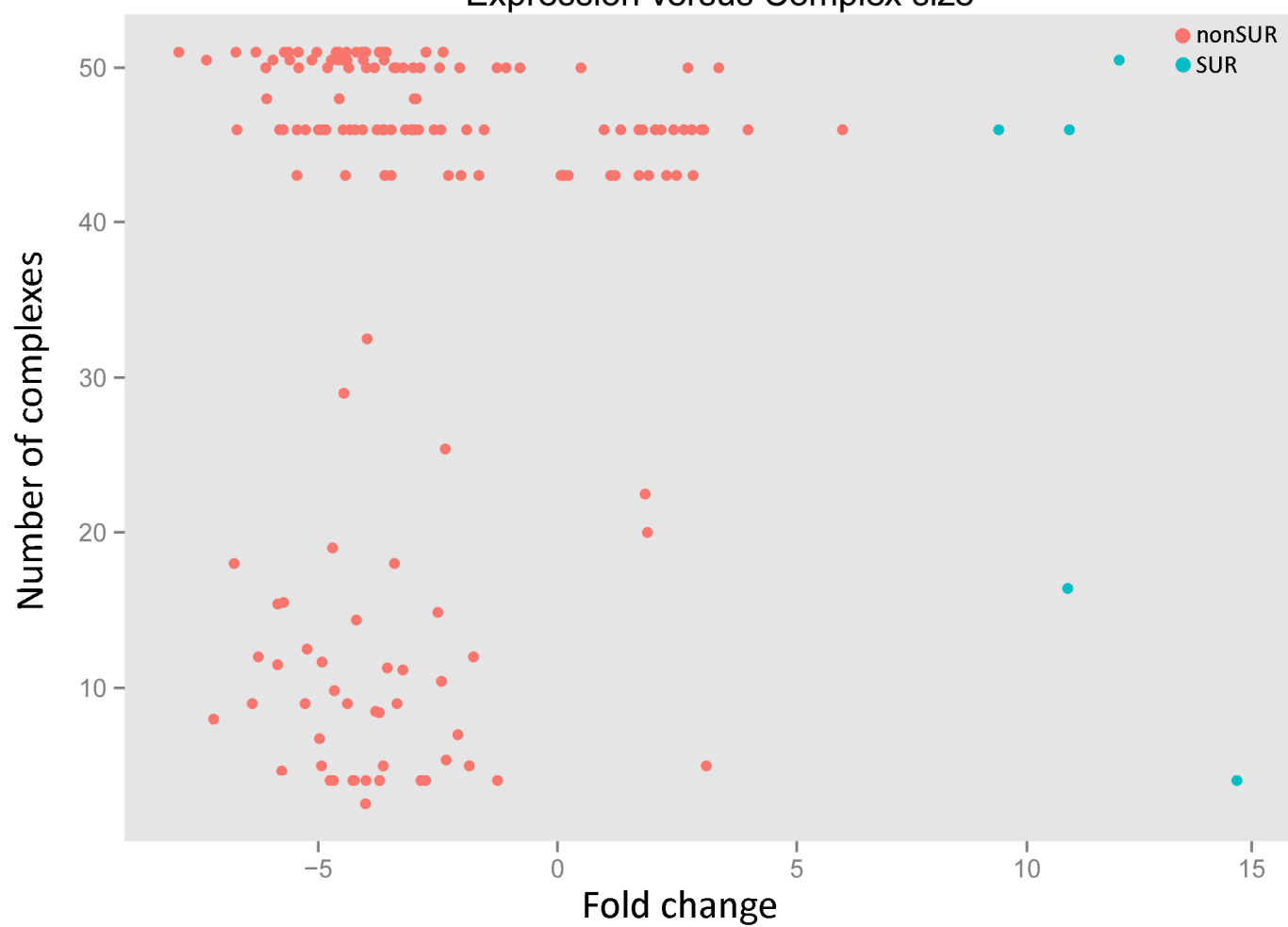

Supplement: Additional file 7: Figure S5 — CORUM complex membership and complex size distribution vs expression for strongly upregulated (SUR) and non-strongly upregulated (non-SUR) RNA-binding proteins. No trends were observed when comparing the CORUM characteristics with expression. [file gb-2014-15-1-r14-S7.pdf]
