# Supplementary material for: Dissecting the expression landscape of RNA-binding proteins in human cancers
Source: Genome Biol. 2014 Jan 10;15(1):R14. doi: 10.1186/gb-2014-15-1-r14 (PMC4053825; doi:10.1186/gb-2014-15-1-r14)
Supplement: Additional file 8: Figure S6 — Heat map showing the variation in expression level measured as median absolute deviation (MAD) values for SUR RNA-binding proteins for nine types of cancer. [file gb-2014-15-1-r14-S8.pdf]

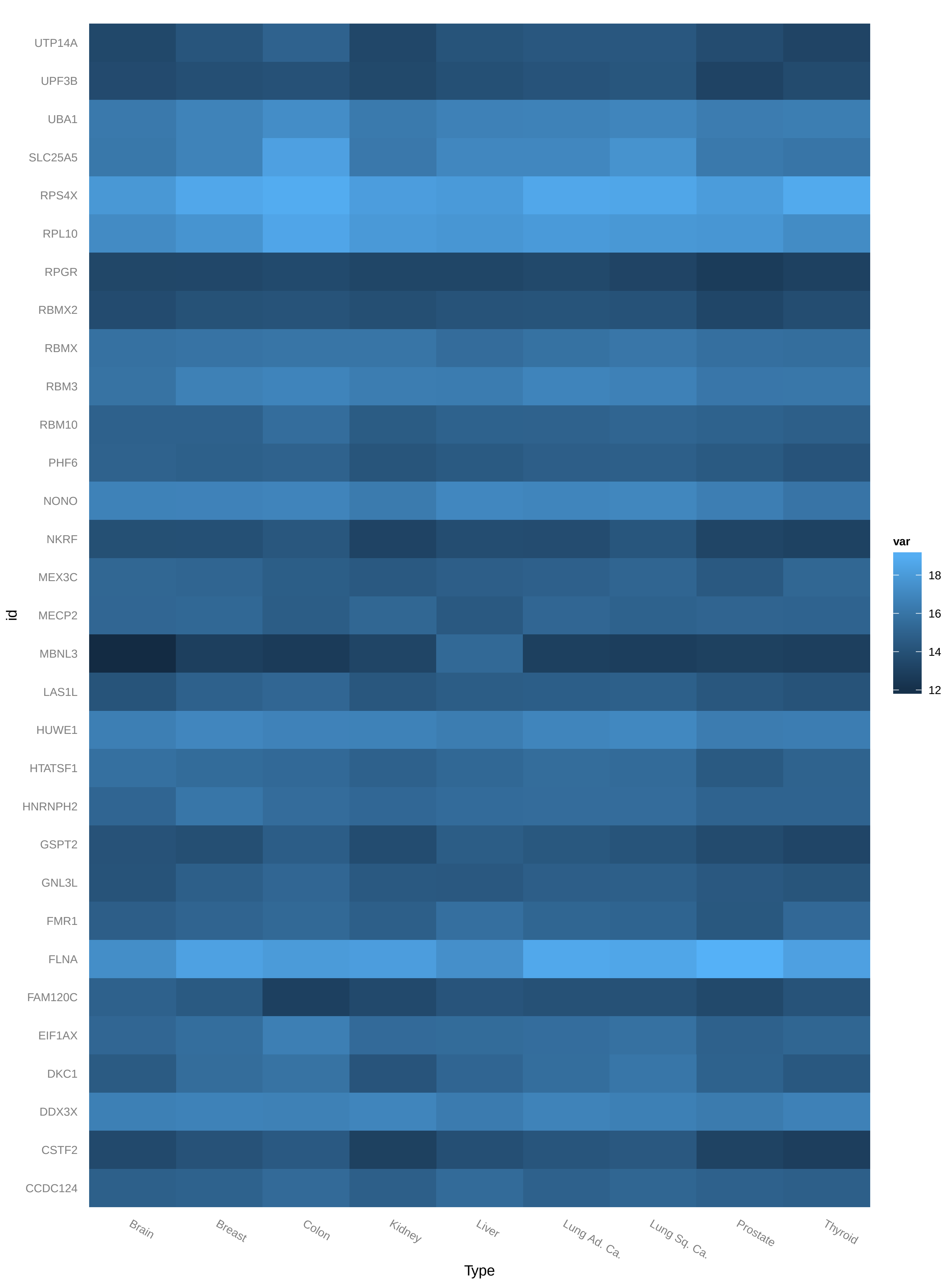
UTP14A
UPF3B
UBA1
SLC25A5
RPS4X
RPL10
RPGR
RBMX2
RBMX
RBM3
RBM10
PHF6
NONO
NKRF
MEX3C
MECP2
MBNL3
LAS1L
HUWE1
HTATSF1
HNRNPH2
GSPT2
GNL3L
FMR1
FLNA
FAM120C
EIF1AX
DKC1
DDX3X
CSTF2
CCDC124
id
Brain
Breast
Colon
Kidney
Liver
Lung Ad. Ca.
Lung Sq. Ca.
Prostate
Thyroid
Type
var
18
16
14
12
